# Taxonomy and Functional Diversity in the Fecal Microbiome of Beef Cattle Reared in Brazilian Traditional and Semi-Intensive Production Systems

**DOI:** 10.3389/fmicb.2021.768480

**Published:** 2021-12-08

**Authors:** Patricia Spoto Corrêa, Carolina Rodriguez Jimenez, Lucas William Mendes, Caroline Rymer, Partha Ray, Luciana Gerdes, Vagner Ovani da Silva, Elisabete Aparecida De Nadai Fernandes, Adibe Luiz Abdalla, Helder Louvandini

**Affiliations:** ^1^Laboratory of Animal Nutrition, Center for Nuclear Energy in Agriculture, University of São Paulo, São Paulo, Brazil; ^2^Laboratory of Molecular Cell Biology, Center for Nuclear Energy in Agriculture, University of São Paulo, São Paulo, Brazil; ^3^Department of Animal Sciences, School of Agriculture, Policy and Development, University of Reading, Reading, United Kingdom; ^4^Reference Laboratory on Classification and Evaluation of Animal Products, Institute of Zootechnics, Nova Odessa, Brazil; ^5^Radioisotopes Laboratory, Center for Nuclear Energy in Agriculture, University of São Paulo, São Paulo, Brazil

**Keywords:** fecal microbiome, metagenome, minerals, beef cattle, production system

## Abstract

The importance of beef production for economy of Brazil and the growing demand for animal protein across the globe warrant an improvement in the beef production system. Although most attention has been on modulation of the rumen microbiome to improve ruminant production, the role of the lower gut microbiome in host health and nutrition remains relatively unexplored. This work aimed to investigate the taxonomy and functional variations in the fecal microbiome of Brazilian beef cattle reared in two different production systems using a metagenomic approach. Sixty male beef cattle from six farms representing semi-intensive (I, *n* = 2) and traditional (T, *n* = 4) Brazilian beef production systems were enrolled in the study. Shotgun sequencing was used to characterize taxonomic and functional composition and diversity of the microbiome in fecal samples collected from each animal. Fecal samples were analyzed for copper (Cu), lead (Pb), nitrogen (N), phosphorous (P), selenium (Se), and zinc (Zn) and stable isotopes of carbon (^13^C) and nitrogen (^15^N). The fecal microbiome was influenced by the beef production systems with greater functional and lower taxonomic diversity in beef cattle feces from I systems compared with that from T systems. The concentration of N, P, and Zn was higher in beef cattle feces from I systems compared with that from T systems and was associated with taxonomic and functional profile of fecal microbiome in I system, suggesting the role of fecal nutrients in shaping system-specific microbiome. Semi-intensive management practices led to a more complex but less connected fecal microbiome in beef cattle. The microbial community in beef cattle feces from I systems was characterized by greater abundance of beneficial bacteria (phylum Firmicutes and butyrate-producing bacteria family *Lachnospiraceae* and genera *Anaerostipes*, *Blautia*, *Butyrivibrio*, *Eubacterium*, *Roseburia*, and *Ruminococcus*). In addition, the fecal abundance of microbial genes related to immune system, nutrient metabolism, and energy production was greater in beef cattle raised under I systems compared with that under T systems. Findings of the current study suggest that semi-intensive management practices could facilitate the development of a healthier and more efficient fecal microbiome in beef cattle by driving an increase in the abundance of beneficial bacteria and functional genes.

## Introduction

Brazil is the largest beef exporter in the world with an annual export of 2.49 million tons of carcass weight in 2019 (ABIEC, 2020; USDA, 2020). In addition, beef production is an important driver of the economy of Brazil, accounting for 8.5% of total gross domestic product of Brazil in 2018 (ABIEC, 2020). The predominant breed found in more than 90% of commercial beef herds in Brazil is the Nelore (ABIEC, 2020). This breed is resilient in the Brazilian environment, and most importantly, it is well adapted to the traditional pasture-based beef production system. However, in recent years, the adoption of intensive and semi-intensive production systems has increased, mainly due to pressure to scale up the production in response to the growing protein demand for human consumption and secondarily to the decline pasture land in Brazil (Maciel et al., 2019; Vale et al., 2019). The major difference in management practices between traditional and semi-intensive production systems is that large amounts of concentrates and feed supplements are used in semi-intensive systems. In addition, pasture is well managed in semi-intensive systems to provide cattle access to high-quality forages. Many studies have explored the relationship between cattle production system, dietary components, and the resident gut microbiota (Mu et al., 2019; Qiu et al., 2019; Andrade et al., 2020; Freetly et al., 2020). Most studies focused on the ruminal microbiota, which is understandable considering their substantial contribution to the generation of substrates for energy and protein supply to the host. However, the lower gut microbiota play an important role in animal health and immunity and contribute, to a lesser extent, to energy supply to the host (Zhang et al., 2019; O’Hara et al., 2020; Valerio et al., 2020). Therefore, it is critical to characterize the fecal microbiota to understand their contribution to host health and nutrition and to understand the effect of management system on the fecal microbiota composition.

In the recent years, there has been an increasing acknowledgment of the need to examine host-microbiome interactions in the rumen and in the lower gut to fully exploit the potential of the gut microbiome for sustainable ruminant production (Hagey et al., 2019; O’Hara et al., 2020). In addition, feces has been used as a proxy of the gut microbiota in animals because of the non-invasive nature of fecal sampling (Muiños-Bühl et al., 2018; Yan et al., 2019; Andrade et al., 2020). A few studies have investigated the association between fecal microbiota and animal phenotypes in Brazilian beef cattle (de Oliveira et al., 2013; Bessegatto et al., 2017; Lopes et al., 2019; Andrade et al., 2020), but there has been little investigation of changes in the microbiota in feces of beef cattle raised under different production systems.

Although investigation of the microbiota provides useful information about the composition of the microbial community, this does not of itself provide a meaningful insight into functional changes in the microbiota. Therefore, we investigated the fecal microbiome by characterizing the functional metagenome and the composition of microbial communities in the feces of beef cattle raised under semi-intensive (fed concentrate supplement) or traditional (without concentrate supplement feeding) management systems. Because the diet plays a key role in manipulating the composition and metabolism of the gut microbiota and the Brazilian beef production systems are distinguished primarily by their difference in dietary management (Myer et al., 2017; Vale et al., 2019), we hypothesized that beef cattle raised under semi-intensive and traditional production systems will show differences in their fecal microbiome. The objective of this study was to determine the taxonomic and functional variations in the fecal microbial communities seen across the beef production systems using a metagenomic approach. Such investigation will provide further insight into the association between management practices and ruminant production and health mediated by changes in the composition and function of the gut microbiota.

## Materials and Methods

### Selection of Beef Farms and Animals

Standard practices of animal management and sampling procedures were approved by the Ethics Committee on the Use of Animals of the Center for Nuclear Energy in Agriculture, University of São Paulo, Piracicaba, Brazil, under protocol no. 007-2018. Six farms from the northwest region of São Paulo, Brazil, were recruited in this study and were representative of semi-intensive (I; Farms 1 and 2, *n* = 2) and traditional (T; Farms 3–6, *n* = 4) Brazilian beef production systems. This classification was based on the practice of feeding supplements (silages and concentrates), rotational grazing, and pasture fertilization by the nominally semi-intensive systems. Feed and nutrient composition of the diets are presented in Supplementary Table 1. Although Farm 5 adopted the practice of feeding supplements, it was categorized as a traditional production system because the amount of daily supplement feeding was very low (300 g per animal).

### Sample Collection and Feed Analysis

On each farm, groups of animals in the same health conditions (no history and symptoms of bad health/any disease) were identified by sex (male) and age (young: approximately 12 months old, and adults: 24–28 months old). Within each age group, five animals were randomly selected for feces collection, i.e., 10 animals per farm. Fecal samples (*n* = 60) were collected from the rectum into sterile cryogenic tubes (KASVI, São José dos Pinhais, Brazil) and stored immediately in liquid nitrogen for further molecular analysis. A subset of fecal samples was collected for the analysis of carbon (^13^C) and nitrogen (^15^N) stable isotopes and minerals to trace the feed recently consumed by the animals and frozen in liquid nitrogen. Pasture samples (in triplicate) were collected in the paddock where the animals were grazing, using a 0.25-m^2^ mold cut at a height of 10 cm above the soil surface to represent the available biomass for grazing. Forage samples were immediately stored at 4°C, until processing for analysis of chemical composition. Any weed or shrubs spread within the paddock were identified and the most abundant ones were sampled and stored at 4°C until processing. Samples of supplementary feed and mineral supplements that were offered to the animals were collected as well. All forages, shrubs, and supplements were dried at 40°C until constant weight was achieved. The dry weight of forages was used to determine dry matter (DM) content and thus to estimate the biomass available for grazing at each farm. Dried feed samples were ground through a 1-mm screen and analyzed for residual DM, organic matter (OM), crude protein (CP), neutral detergent fiber (NDF), acid detergent fiber (ADF), and lignin (LIG) according to the established methods of the Association of Official Analytical Chemists (AOAC, 2011).

### Carbon and Nitrogen Stable Isotopes, Minerals, and Inorganic Phosphorous Analysis in Feces

For δ^13^C and δ^15^N evaluation, fecal samples were dried and ground through a 0.2-mm screen and homogenized, and a subsample transferred to a tin capsule (code D1008 and size of 8 × 5 mm). Total C, N, and isotopic composition (^13^C and ^15^N) were determined in an elemental analyzer (CHN-1110, Carlo Erba, Rodano, Italy) interfaced to an isotope ratio mass spectrometer (Delta Plus, ThermoFisher Scientific, Bremen, Germany). The concentration of C and N was expressed in percent, and the stable isotopic ratio was expressed in the classical “δ” notation according to equation (Craig, 1953): δ*^n^X* = (*R*_sample_/*R*_standard_ – 1) × 1,000 (1), where *X* is C or N; *n* is the mass number of the heavier isotope (^13^C or ^15^N); and *R*_sample_ and *R*_standard_ are the isotopic ratio of the samples and the standard, respectively. Pee Dee Belemnite for C and atmospheric air for N were used as the primary standards. Minerals [arsenic (As), copper (Cu), zinc (Zn), lead (Pb), and selenium (Se)] were determined in feces using a triple quadrupole inductively coupled plasma mass spectrometer (TQ-ICP-MS, Agilent 8900 series). Briefly, the samples were microwave-assisted acid digested (Milestone ETHOS UP). Isotopes ^75^As, ^63^Cu, ^66^Zn, ^208^Pb, and ^78^Se were monitored to quantify As, Cu, Zn, Pb, and Se mass fractions. Internal standard solution (Agilent Part Number 5188–6525) containing Sc (m/z = 45), Ge (m/z = 72), and Rh (m/z = 103) was used to correct the plasma stability. SRM 1643d Trace Elements in Water (NIST) was used for analytical quality control. For determination of inorganic P, feces were weighed in a precise scale and DM and ash were determined. The ash residue is digested, filtered, and then P-quantified by colorimetry, using the ammonium molybdate-vanadate method, as described by Sarruge (1974).

### Fecal DNA Extraction and Sequencing

Total DNA was extracted from freeze fecal samples (0.25 g) using the DNeasy ^®^ PowerLyzer ^®^ PowerSoil ^®^ kit (Qiagen, Hilden, Germany) according to the protocol of the manufacturer. The quality of extracted DNA was checked by 0.8% agarose gel electrophoresis and NanoDrop 8,000 spectrophotometer (Thermo Fisher Scientific, Waltham, United States). Qubit 2.0 fluorometer (Invitrogen, Carlsbad, United States) was used to measure the concentration of extracted DNA. In total, 60 DNA samples that qualified for library preparation were sequenced using the Illumina HiSeq 2500 platform [2 × 150 base pairs (bp)] (Illumina, Inc., San Diego, United States) at Novogene Corporation, Inc. (U.S. Subsidiary and UC Davis Sequencing Center).

### Shotgun Metagenomic Data Processing

Approximately 1.5 billion sequences were obtained from samples using the shotgun metagenomic approach, with an average of 22–32 million sequences per sample. Raw sequences were filtering to discard those with low-quality bases (quality score < 20) under default parameters using HiSeq software (Illumina). Sequences were paired using software PEAR, a paired-end read merger (Zhang et al., 2014), and low-quality bases with quality score < 20 and nucleotides < 50 bp were removed. The remaining sequences were than uploaded and annotated in the Metagenomics Rapid Annotation (MG-RAST) pipeline version 3.3.3.3 (Meyer et al., 2008). Taxonomic profile was generated by matching the normalized sequence to the RefSeq database (O’Leary et al., 2016), and the database and infrastructure for comparative genomics (SEED) (Aziz et al., 2008) and Kyoto encyclopedia of genes and genomes (KEGG) (Kanehisa and Goto, 2000) databases were used to determine functional profile (default parameters, minimum alignment length of 50 bp, minimal identity of 60%, and an *E*-value cutoff of *E* < 1 × 10^–5^). The matrices generated about taxa and potential functions were exported and used for statistical analyses. The metagenome data are available at MG-RAST server under the project “Cattle fecal metagenome” (ID mgp89663).

### Statistical Analyses

Alpha diversity was calculated on the basis of the number of observed taxa or function (richness) and the Shannon diversity index. The means were compared by Tukey *t*-test, and *P* ≤ 0.05 was considered statistically significant. Both analyses were performed on PAST v3 software (Hammer et al., 2001), using a matrix of abundance at the genus level affiliated to the RefSeq database. Principal components analysis (PCA) was performed to visualize the microbial community structure and function profile among cattle with different ages (adult and young). Redundancy analysis (RDA) was used to visualize the microbial community and function structure from both systems and to determine its correlation with nutrient composition of feed, nitrogen stable isotopes, minerals, and phosphorus in feces. First, the matrices were analyzed using detrended correspondence analysis (DCA) to evaluate the gradient size of the genus distribution, which indicated linearly distributed data (length of gradient < 3), suggesting the RDA as the best-fit mathematical model for the data. Forward selection and the Monte Carlo permutation test were applied with 1,000 random permutations to verify the significance of environmental parameters upon the biological variables. RDA plots were generated using Canoco 4.5 software (Biometris, Wageningen, Netherlands). Permutational multivariate analysis of variance (PERMANOVA) (Anderson, 2001) with Bray–Curtis dissimilarity was used to confirm the difference of the structure and function profile among the ages and systems. To further investigate the correlation between microbial diversity (Shannon index) and fecal parameters, we ran a linear regression in the “ggpubr” R package. The differences in microbial taxonomic and functional composition between production systems were performed in Statistical Analysis of Metagenomic Profiles (STAMP) software, version 3.0 (Parks et al., 2014) using two-sided Welch’s *t*-test (Welch, 1947) followed by the Benjamini–Hochberg false discovery rate correction (Benjamini and Hochberg, 1995). In addition, to explore the complexity of interactions between microbial genus, a network analysis was performed. For this, non-random co-occurrence analysis between microbial genus was performed using SparCC (Friedman and Alm, 2012). For each network, the *P*-values were obtained by 99 permutations of random selections of the data table, being subjected to the same analytical pipeline. The statistically significant (*P* < 0.01) SparCC correlations with a magnitude of > 0.95 or < −0.95 were included into the analysis. The nodes of the reconstructed networks represent microbial genus, whereas the edges represent significantly positive or negative correlations between nodes. The topology of the networks was calculated on the basis of a set of measurements including the number of nodes and edges, modularity, number of communities, average path length, network diameter, average degree, and clustering coefficient (Newman, 2003). The co-occurrence network analysis was carried out using the Python module “SparCC,” and the network reconstruction and properties measurements were calculated using Gephi (Bastian et al., 2009).

## Results

### Fecal Concentration of Carbon and Nitrogen Stable Isotopes and Minerals

Fecal concentration of As and Pb was higher in beef cattle from T systems, whereas the concentration of Zn and P was higher in the feces of beef cattle from I systems (Table 1). Fecal concentration of N (%N) was higher in beef cattle feces from I systems, but %C and C/N values were higher for fecal samples collected from beef cattle raised under T systems (Table 2).

**TABLE 1:** Minerals concentration from cattle feces of I (semi-intensive) and T (traditional) beef production systems.

Variables	I	T	*se*	*P*-value
Zinc (mg/kg DM)	124.3	95.8	10.2	0.0319
Copper (mg/kg DM)	24.5	27.2	3.26	0.5158
Lead (mg/kg DM)	1.07	1.83	0.08	0.0001
Arsenic (mg/kg DM)	0.45	0.75	0.04	0.0001
Phosphorous (g/kg DM)	0.64	0.39	0.21	0.0001
Selenium (mg/kg DM)	−	−	−	−

*Significance determined using the general linear model (GLM). (−) Not detectable.*

**TABLE 2:** Stable isotope values (δ ^13^C, δ ^15^N) and C/N ratio from cattle feces of I (semi-intensive) and T (traditional) beef production systems.

Variables	I	T	*se*	*P*-value
%N	1.75	1.49	0.36	0.0051
%C	33.0	40.0	4.70	0.0002
C/N	18.7	27.7	4.88	0.0001
δ ^15^N	5.00	5.10	1.03	1.1803
δ ^13^C	−14.7	−14.9	0.69	0.6240

*Significance determined using the general linear model (GLM).*

### Microbial Community Structure

**FIGURE 1:**
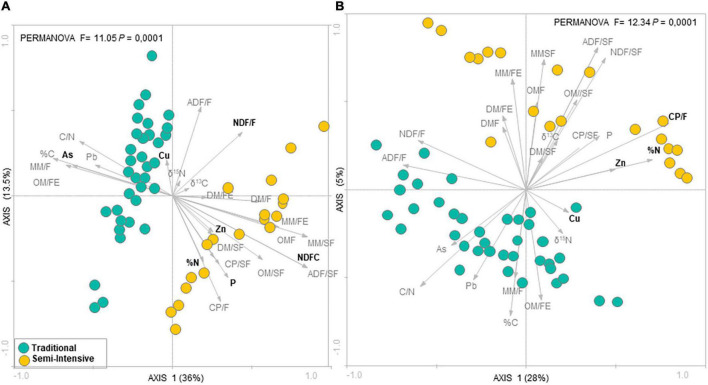
Redundancy analysis (RDA) of the fecal microbial communities at **(A)** genus level and **(B)** functional profile in beef cattle from semi-intensive (yellow) and traditional (blue) systems. Arrows indicate correlation between environmental parameters and community structure. Bold letters indicate significant correlations evaluated via the Monte Carlo permutation test (*P* < 0.05).

According to the RDA analysis, the fecal microbial community in I samples clustered distinctly from T samples for both taxonomic composition (PERMANOVA, *F* = 11.05, *P* = 0.0001) (Figure 1A) and functional profile (PERMANOVA, *F* = 12.34, *P* = 0.0001) (Figure 1B). The RDA followed by Monte Carlo analysis showed that the bacterial community in beef cattle feces from I farms was associated with both forage and supplement NDF content and P for taxonomic composition and with forage CP content for functional profile. In addition, in I system, feed N (%N) and fecal Zn concentrations were associated with both taxonomic composition and functional profile of the fecal microbial community. Whereas, in T system, fecal microbial community was associated with fecal As concentration for taxonomic composition and with fecal Cu concentration for functional profile. In contrast, according to the PCA analysis, there was no difference among ages (PERMANOVA, *P* > 0.05; Supplementary Figures 1, 2).

### Microbial Community Diversity and Composition

**FIGURE 2:**
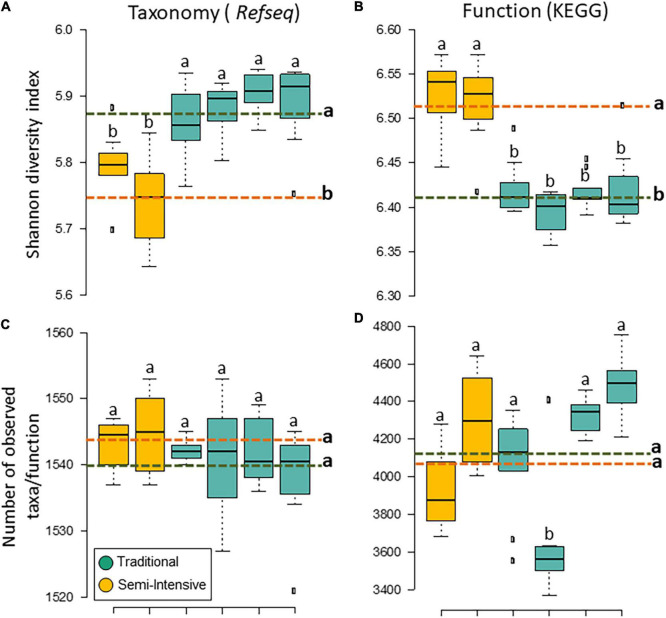
Diversity measurements of microbial communities in the feces of beef cattle reared in semi-intensive (yellow) and traditional (blue) systems. Taxonomic **(A)** diversity and **(C)** richness are based on genera level (RefSeq database), and functional **(B)** diversity and **(D)** richness are based on subsystem level 3 (SEED database). Error bars represent the standard deviation of four independent replicates. Different lower-case letters refers to significant differences between treatments based on Tukey’s honestly significant difference test (*P* < 0.05). The dashed lines represent the average values for each treatment.

**FIGURE 3:**
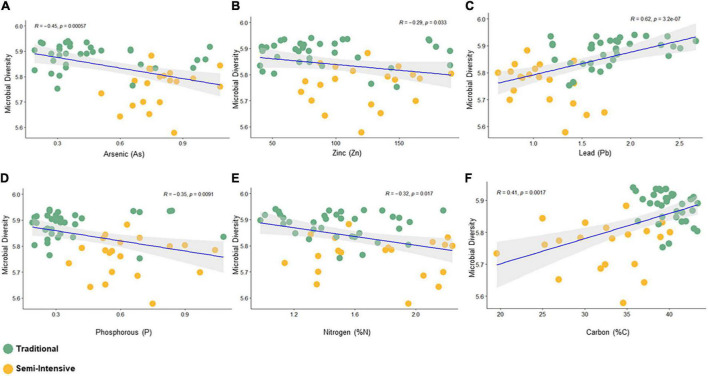
Spearman correlation between bacterial diversity (Shanon index) and fecal parameters (*P* < 0.05) constructed using the R package “corrplot.” **(A)** Arsenic, **(B)** Zinc, **(C)** Lead, **(D)** Phosphorous, **(E)** Nitrogen, and **(F)** Carbon.

**FIGURE 4:**
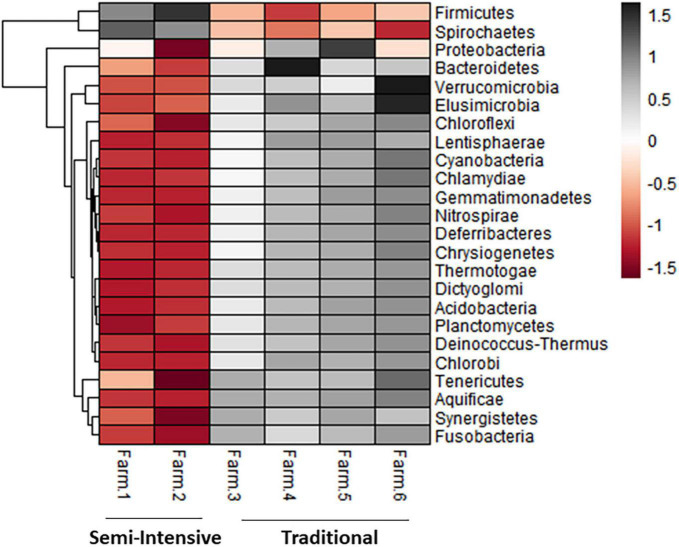
Heatmap showing the differential abundance of bacterial phylum in the fecal microbiota farms from semi-intensive and traditional management systems. The color key relates the heatmap colors to the standard score (z-score), i.e., the deviation from row mean in units of standard deviations above or below the mean.

**FIGURE 5:**
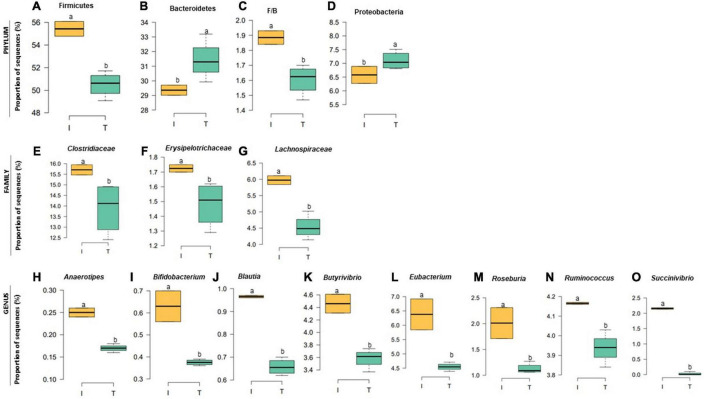
Relative abundance at bacterial phylum **(A–D)**, family **(E–G)**, and genus **(H–O)** in the fecal microbiota of I (semi-intensive) and T (traditional) beef production systems. Different letters (a,b) are significantly different, *P-*value < 0.005.

The taxonomic diversity was lower, whereas functional diversity was higher in the fecal microbiota of I farms compared with T farms (Figures 2A,B). Bacterial richness were similar among the systems (Figures 2C,D). Furthermore, an investigation of the association between mineral elements, C and N, and the bacterial diversity of the fecal samples showed that Zn, P, Pb, As, C, and N were correlated (*P* < 0.05) with fecal bacterial diversity (Figures 3A–F). In the fecal bacterial community, the abundance of 24 phyla was different between I and T farms, and the relative abundance of most taxa was relatively greater in the feces of beef cattle raised under T management system (Figure 4). Interestingly, the phyla, Bacteroidetes (31.5% of the total sequences), and Proteobacteria (7.4%) were more abundant in the feces of beef cattle raised on T farms compared to I farms (Figure 5), whereas the abundance of Firmicutes (53.4%) and Firmicutes-to-Bacteroidetes ratio (F/B) were higher in fecal samples from I farms (Figure 5). Specific microbial groups were enriched by the type of management system. In addition, the abundance of 202 families and 491 genera was influenced by the type of management system (Supplementary Tables 2, 3, *P* < 0.05). Here, we highlight beneficial bacteria, such as the families *Bifidobacteriaceae*, *Clostridiaceae*, and *Erysipelotrichaceae* and genera *Lachnospiraceae* (Figures 5D–G, *P* < 0.05), *Anaerostipes*, *Butyrivibrio*, *Eubacterium*, *Roseburia*, *Ruminococcus*, *Succinivibrio*, and *Blautia* had a higher relative abundance in the feces of cattle reared on I beef farms (Figures 5H–O, *P* < 0.01).

### Microbial Functional Profile

**FIGURE 6:**
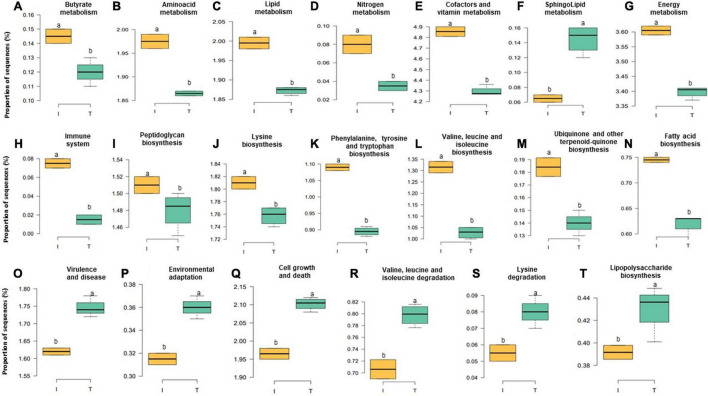
Relative abundance at microbial genesin the fecal microbiota of I (semi-intensive) and T (traditional) beef production systems **(A–T)**. Different letters (a,b) are significantly different, *P-*value < 0.005.

Accompanied with the distinct changes in taxonomic composition, we also observed changes in the fecal functional metagenome composition between I and T farms. In general, we observed a difference between the systems in 106 functional genes (Supplementary Table 4). Here, we highlight the abundance of genes encoding various functions related to nutrient metabolism; energy production; immune system, including biosynthesis of peptidoglycan, phenylalanine, tyrosine and tryptophan, valine, leucine, isoleucine, lysine, fatty acid, ubiquinone, and other terpenoid-quinones; and the metabolism of lipid, butyrate, nitrogen, energy, amino acid, cofactors, and vitamins and sequences related to the immune system, which were relatively higher, in microbial communities of the feces from I beef farms (Figures 6A–E,G–N, *P* < 0.05). In contrast, genes involved in virulence, disease and defense, lipopolysaccharide (LPS) biosynthesis, sphingolipid metabolism, cell growth and death, environmental adaptation, and the degradation of valine, leucine, isoleucine, and lysine that were more abundant, in the feces from T farms (Figures 6F,O–T, *P* < 0.05).

### Co-occurrence Network Analysis

**FIGURE 7:**
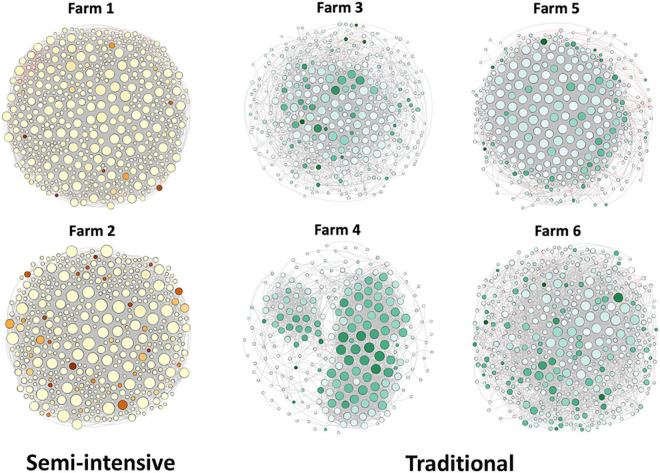
Network co-occurrence analysis of microbial communities in beef cattle feces from semi-intensive and traditional management system farms. Each node represents taxa affiliated at genus level (based on shotgun), and the size of node is proportional to the number of connections, i.e., degree. Each node was labeled at phylum level.

To explore the interaction between the fecal microbial communities within the two different management systems, we reconstructed co-occurrence networks at the genus level on the basis of the metagenome data (Figure 7). In general, the profile of microbial network exhibited variations among the farms. The microbial communities in the feces of beef cattle reared in I had a higher number of microbial groups with a greater number of interactions between them (Table 3). However, fecal microbial communities from I systems had a lower average clustering coefficient compared to T systems. On the other hand, fecal microbial networks in T systems had a smaller network diameter and shorter average path length compared to I systems.

**TABLE 3:** Topological properties of the networks as inferred by SparCC of I (semi-intensive) and T (traditional) beef production systems.

Network properties	Farm 1	Farm 2	Farm 3	Farm 4	Farm 5	Farm 6
Number of nodes^a^	552	427	389	355	227	447
Number of edges^b^	5,567	2,840	1,768	4,856	2,461	1,911
Positive edges^c^	4,921	2,821	1,753	4,756	2,460	1,877
Negative edges^d^	646	19	15	100	1	34
Modularity^e^	0.829	0.684	0.820	0.527	0.574	0.811
Number of communities^f^	35	43	29	34	22	39
Network diameter^g^	18	32	17	12	6	20
Average path length^h^	6.293	11.95	5.597	4.485	1.922	6.308
Average degree^i^	20.17	13.30	9.09	27.35	21.68	8.55
Average clustering coefficient^j^	0.612	0.675	0.694	0.704	0.771	0.638

*^a^Microbial taxon (at genus level) with at least one significant (P < 0.01) and strong (SparCC > 0.9 or < -0.9) correlation.*

*^b^Number of connections/correlations between nodes obtained by SparCC analysis.*

*^c^SparCC-positive correlation (>0.9 with P < 0.01).*

*^d^SparCC-negative correlation (<-0.9 with P < 0.01).*

*^e^The capability of the nodes to form highly connected communities, that is, a structure with high density of between nodes connections (inferred by Gephi).*

*^f^A community is defined as a group of nodes densely connected internally (Gephi).*

*^g^The longest distance between all possible pairs of nodes in the network, measured in number of edges (Gephi).*

*^h^Average network distance between all possible pairs of nodes or the average length off all edges in the network (Gephi).*

*^i^The average number of connections per node in the network, that is, the node connectivity (Gephi).*

*^j^How nodes are embedded in their neighborhood and the degree to which they tend to cluster together (Gephi).*

## Discussion

The present study, to our knowledge, was the first to compare the cattle fecal microbiome from Brazilian traditional and semi-intensive beef production systems. The bovine fecal microbiota is complex, and management practices play an integral role in shaping the establishment of the fecal microbial community (Shanks et al., 2011). The data from the current study showed that the management system had a significant impact on shaping the beef cattle fecal microbiome diversity and structure. Unlike other studies, age of animals did not influence the fecal microbiome in the current study. This discrepancy was likely because young animals recruited in the current study were relatively more mature (12 months old) compared to other studies that reported age-driven changes in the fecal microbiome (Oikonomou et al., 2013; Klein-Jöbstl et al., 2014). In the current study, I farms clustered distinctly from T farms for both taxonomic and functional structure of the fecal microbial communities, showing the difference in fecal microbiome between the production systems differed in feeding management. Difference in the structure of bacterial communities from feces of cattle fed different diets has been reported before by Kim et al. (2014) and Azad et al. (2019). In the current study, relatively low taxonomic diversity (Shannon diversity index) in the microbial community of fecal samples from I beef farms was consistent with a recent finding that fecal bacterial diversity in growing cattle decreased as dietary concentrate and energy increased (Zhang et al., 2018; Qiu et al., 2019). Similarly, in a recent study, the diversity and structure of the rumen and fecal microbiota in cattle responded differently to dietary treatments (Azad et al., 2019). Alpha-diversity indices of rumen microbial communities have also been reported to contribute to the variation in feed efficiency of cattle, where inefficient animals possessed more diverse microbial communities (Li et al., 2019a). Interestingly, in the current study, relatively less diverse bacterial diversity in the feces from I farms had highly diverse functional profile, suggesting more efficient microbiome in the gut of cattle reared in I management systems. This conclusion could be supported by recent findings that the rumen microbiome of more efficient cattle had more diverse functional potential and higher activities compared with that of less efficient animals (Shabat et al., 2016; Li and Guan, 2017). Similarly, Li et al. (2019a) reported that rumen microorganisms of inefficient cattle produce more products that can be harmful or of little use, whereas relatively simple and less active microbiota in the rumen of efficient animals produced fewer but more useful fermentation products that were absorbed more efficiently by the host.

In addition, the correlation of the fecal microbiome with CP, NDF, and %N on I farms and with %C on T farms suggested a strong influence of the feed composition on the fecal microbiome. Relatively higher concentration of CP and NDF in forages and supplements used on I farms compared with that on T farms (63 and 788 g/kg DM vs. 51 and 737 g/kg DM, CP, and NDF, respectively) indicates higher nutrient intake by the cattle reared in I systems, with potentially better synchronization of energy and N availability for the fecal microbiota that are essential for the fermentation process.

In the current study, relatively higher abundance of the phylum Firmicutes but lower abundance of the phylum Bacteroidetes in the beef cattle from I farms suggested the possibility of beef cattle reared in I systems being more efficient in utilizing nutrients. Díaz Carrasco et al. (2017) suggested that increased F/B can improve performance in bovines, citing previous studies that reported a correlation between F/B and phenotypic parameters of performance such as body weight gain and milk fat yield (Jami et al., 2014; Myer et al., 2015). In addition, increased abundance of the genus *Succinivibrio* in the gut of possibly more efficient beef cattle reared in I system is consistent with another report evaluating the gut microbiota and feed efficiency in cattle (Li et al., 2019b). Accompanied with a shift in the taxonomic composition, the functional profile of the fecal microbiota in beef cattle from I farms differed from that T farms with greater abundance of gene coding for energy production and nutrient metabolism including biosynthesis of amino acids, ubiquinone, and other terpenoid-quinone. Nutritionally essential amino acids synthesized *de novo* by the gut microbiota work as potential regulatory factors in amino acid homeostasis and gut health (Lin et al., 2017), suggesting that the gut of beef cattle reared in I systems harbored a relatively more efficient microbiome characterized by enriched gene coding for amino acid synthesis. Increased abundance of gene coding for ubiquinone and other terpenoid-quinone in beef cattle feces from I farms further suggested that beef cattle reared on I farms were possibly more efficient in utilizing energy because high producing cows have been previously found to have upregulated metabolic pathways of ubiquinone and other terpenoid-quinone biosynthesis (Mu et al., 2019).

In addition to the dietary factors mentioned above, it is likely that the fecal microbiome in beef cattle was influenced by dietary mineral supply, as indicated by a significant correlation between the microbial diversity and fecal mineral concentrations observed in the current study. Considering that feces can be a bioindicator of dietary nutrient supply, which has proven to be a cost-effective and reliable method in studies of ruminants (Showers et al., 2006; Fredin et al., 2014; Yang et al., 2020), relatively higher fecal concentrations of P and Zn in I farms and As and Pb in T farms suggested that the dietary supply of these minerals to beef cattle was higher in respective farms, which might have contributed to the difference in fecal microbial community structure between I and T management systems. Indeed, it is widely known that minerals and trace elements are essential micronutrients and an important driver of gut microbiota modulation (Skrypnik and Suliburska, 2018; Chang et al., 2020; Yang et al., 2020). In addition, hematological and biochemical parameters from the same animals used in the current study were evaluated by Jimenez et al. (Unpublished^1^). Although hematological parameters were not influenced by the production system, blood urea concentration was higher in beef cattle reared on I farms, and blood glucose concentration was higher in beef cattle reared on T farms. This indicates that differences that were observed in the microbiome were not reflected in the hematological response of the animals, but there were differences in blood biochemistry, which would be expected as a result of the differences in the diet.

Our analysis also showed that specific fecal nutrients were correlated with bacterial diversity. In the current study, bacterial diversity in the feces from I farms was negatively correlated with fecal P and Zn concentrations. However, this reduced diversity could be explained by increased abundance of specific groups of beneficial bacteria (family *Lachnospiraceae* and genera *Anaerostipes*, *Blautia*, *Butyrivibrio*, *Eubacterium*, *Roseburia*, and *Ruminococcus*) in I farms. All these bacteria are known to produce butyrate in the gut (Li et al., 2012; Levy et al., 2016; Geirnaert et al., 2017), providing a source of energy for the mucosa and hence promoting epithelial health (Kim et al., 2014). Increased dietary phosphorus supply has been related to an increase of butyrate-producing bacteria in the cecal digesta of broiler chickens (Borda-Molina et al., 2016). Similarly, Zn is an important micronutrient that promotes the growth of beneficial gastrointestinal bacteria responsible for maintaining epithelial integrity (Chang et al., 2020; Yang et al., 2020). In addition, the higher abundance of *Bifidobacterium* in I samples suggested a healthier microbiota in the gut of beef cattle reared in I systems, because this microorganism has been considered both a probiotic and symbiont (Feng et al., 2018) and was associated with healthy fecal microbiota in dairy calves (Gomez et al., 2017).

Interestingly, the most abundant class abovementioned belongs to the phylum Firmicutes. An increase in the abundance of Firmicutes in the gut of semi-intensively managed beef cattle with possibly high dietary supply of Zn was in accordance with the finding by Chang et al. (2020), who reported a higher abundance of Firmicutes in the fecal microbiota of newborn dairy calves supplemented with Zn. In addition, it has been reported that Zn is a gatekeeper of immune function (Wessels et al., 2017) and gut microbiota play a key role in host immunostasis (Lazar et al., 2018). Interestingly, functional analysis showed that the fecal bacterial community in beef cattle reared on I farms had a higher abundance of sequences related to the biosynthesis of peptidoglycan and immune system, which are important for generating innate immune memory and the defense mechanism in bacteria (Horvath and Barrangou, 2010; Negi et al., 2019).

In the current study, the microbiota in beef cattle feces from T farms was characterized by an increased abundance of the phylum Proteobacteria, which has been associated with diet change, gut dysbiosis, or enrichment of stress-response genes in the gut microbiota of cattle (Auffret et al., 2017). Increased abundance of Proteobacteria in parallel to reduced abundance of Firmicutes in the feces from T farms could be attributed to lower fecal concentration of Zn and hence lower dietary supply of Zn to beef cattle on T farms that are used as indicator of gut dysbiosis (Reed et al., 2015; Wu et al., 2019). Therefore, a lower dietary supply of Zn to animals, which was possibly the case on T farms, may contribute to the disruption of gut microbiota homeostasis.

In addition to the abundance of Proteobacteria, genes related to virulence, disease and defense, LPS biosynthesis, sphingolipid metabolism, cell growth and death, and environmental adaptation were more abundant in the fecal microbiome of beef cattle from T farms. Genes related to virulence, disease and defense, or stress response have been found to be associated with an increased abundance of Proteobacteria (Auffret et al., 2017). An enrichment of genes related to the biosynthesis of LPSs in feces from T farms can be explained by an increased abundance of Proteobacteria because all Proteobacteria are Gram-negative bacteria and Gram-negative bacteria contain LPS in their outer membrane (Cani et al., 2007). LPS is a potent endotoxin and has been associated with gut disorders and disease in the host (Shin et al., 2015; Plaizier et al., 2018).

Relatively high fecal concentrations of As and Pb in beef cattle reared on T farms suggested that the gut microbiota in these cattle were exposed to high concentrations of these heavy metals. Although oral administration of a high concentration of As resulted in the enrichment of genes related to LPS biosynthesis in the gut microbiome of mice (Chi et al., 2017), the exposure to a high concentration of Pb leads to a perturbation in the gut microbiota characterized by a reduced abundance of beneficial bacterial genera (Gao et al., 2017). Therefore, in beef cattle reared on T farms, the exposure of the gut microbiota to high concentrations of heavy metals might have contributed to relatively poor gut health or development of a microbiota that is prone to dysbiosis. However, it is difficult to attribute the difference in fecal concentrations of As and Pb between I and T systems to different dietary management practices because the exposure of beef cattle to these heavy metals can be through the consumption of soil, water, and air (Johnsen and Aaneby, 2019). In addition, the concentrations of heavy metals reported in the current study are very low compared to their toxic doses, and therefore, these heavy metals might not have contributed to the gut microbiota modulation as much as they would at higher doses (Reis et al., 2010).

The effect of the I and T production systems on microbiome dynamics was assessed using the co-occurrence network analysis, which has been reported to be useful to visualize the impact of different environmental factors on the adaptability of the microbiome (Wang et al., 2019). A comparison of the complexity of interactions between microbes within each production system revealed that the microbial community in the gut of beef cattle reared in I systems had a greater number of microbial groups interacting, but with less connectivity among the microbes (Table 3). Our observation is based on the higher number of nodes and edges found in I farms compared with that in T farms. However, the microbial networks of the I farms had a lower clustering coefficient, which is a measure of the degree to which nodes tend to cluster together, revealing a closer network. This lower connectivity can also be observed in the higher number of communities in the I farm and higher modularity. A more modular structure is characterized by the presence of different groups of nodes (i.e., communities) with some independencies between groups (Newman, 2006). The higher number of communities suggests an increased diversity in species roles and functionality, increasing niche overlap (Poudel et al., 2016). Besides, we observed a lower average path length in T farms. This network property is defined as the average number of steps along the shortest paths between each node, being a measure of efficiency of a network (Zhou et al., 2010; Mendes et al., 2018). Our finding was consistent with the finding of a previous work (Wolff et al., 2017) that reported complex and variable changes in the metabolic networks of the ruminal microbiome as a result of dietary changes. Another study with cows fed corn silage, grass silage, or grass hay diet also provided a deeper insight into how different diets and microenvironments influence the complicated network of bacterial interactions and adaptations (Deusch et al., 2017). In our study, our network analysis revealed that the microbial community was affected by semi-intensive system, increasing the number of groups interacting within the community but with less connectivity among them. This shift in the community dynamics between the two production systems has a potential effect on metabolic pathways within this microbiome.

## Conclusion

Metagenomic investigation of the fecal microbiome in Brazilian beef cattle revealed that some microbial taxa and their genes differ substantially between semi-intensive and traditional production systems with lower taxonomic but greater functional diversity in the fecal microbiome of beef cattle reared in the semi-intensive system. In intensively managed beef cattle, the fecal microbiome was characterized by an increased abundance of beneficial bacteria including butyrate-producing bacteria and microbial genes related to nutrient metabolism, energy production, and immune system. Many dietary and fecal parameters were associated with both taxonomic and functional diversity of the fecal microbiome, but further work is needed to evaluate causative relationships between them. Together, our study findings suggest that the Brazilian semi-intensive management system could promotes a healthier and more efficient gut microbiome than the traditional system, which was accompanied by alterations in fecal microbial community *via* driving an increase in the abundance of beneficial bacteria and functional genes and supporting the development of a complex microbiome.

## Data Availability Statement

The datasets presented in this study can be found in online repositories. The names of the repository/repositories and accession number(s) can be found below: https://www.mg-rast.org/mgmain.html?mgpage=search&search=mgp89663, mgp89663.

## Ethics Statement

The animal study was reviewed and approved by Ethics Committee on the Use of Animals (CEUA) of the Center for Nuclear Energy in Agriculture, University of São Paulo (CENA/USP), Piracicaba, Brazil. Written informed consent was obtained from the owners for the participation of their animals in this study.

## Author Contributions

HL, CR, PR, and AA: conceptualization, validation, supervision, and funding acquisition. PC: performed the data analysis and writing original draft. CJ: performed the laboratory work and writing. LM: performed bioinformatics analysis. LG and VS: collected the samples. ED: contributed to lab analysis. All authors read and approved the final version.

## Conflict of Interest

The authors declare that the research was conducted in the absence of any commercial or financial relationships that could be construed as a potential conflict of interest.

## Publisher’s Note

All claims expressed in this article are solely those of the authors and do not necessarily represent those of their affiliated organizations, or those of the publisher, the editors and the reviewers. Any product that may be evaluated in this article, or claim that may be made by its manufacturer, is not guaranteed or endorsed by the publisher.

## Footnotes

1 Jimenez, C. R., Corrêa, P. S., Lemos, L. N., Rymer, C., Ray, P., Tonks, A., Gerdes, L., Abdalla, A. L., Louvandini, H. (2021). Antibiotic resistance genes in the Brazilian beef cattle management system. Unpublished manuscript.

## Abbreviations

ADF: acid detergent fiber
As: Arsenic
CP: crude protein
Cu: copper
DCA: detrended correspondence analysis
DM: dry matter
I: semi-intensive system
MM: mineral matter
NDF: neutral detergent fiber, OM, organic matter
Pb: lead
PERMANOVA: permutational multivariate analysis of variance
RDA: redundancy analysis
SE: standard error
Se: selenium
STAMP: Statistical Analysis of Metagenomic Profiles
T: traditional system
Zn: Zinc.
