# Supplementary material for: Taxonomy and Functional Diversity in the Fecal Microbiome of Beef Cattle Reared in Brazilian Traditional and Semi-Intensive Production Systems
Source: Front Microbiol. 2021 Dec 8;12:768480. doi: 10.3389/fmicb.2021.768480 (PMC8692951; doi:10.3389/fmicb.2021.768480)
Supplement: Supplementary file 1 [file Data_Sheet_1.pdf]

Frontiers in Microbiology, Supplementary material

Taxonomy and functional diversity in the fecal microbiome of beef cattle reared in Brazilian traditional and semi-intensive production systems

Patricia Spoto Corrêa, Carolina Rodriguez Jimenez, Lucas William Mendes, Caroline Rymer, Partha Ray, Luciana Gerdes, Vagner Ovani da Silva, Elisabete Aparecida De Nadai Fernandes, Adibe Luiz Abdalla, Helder Louvandini

*Corresponding author: Partha Ray E-mail: partha.ray@tnc.org

Running title: Beef cattle fecal microbiome in Brazilian production systems

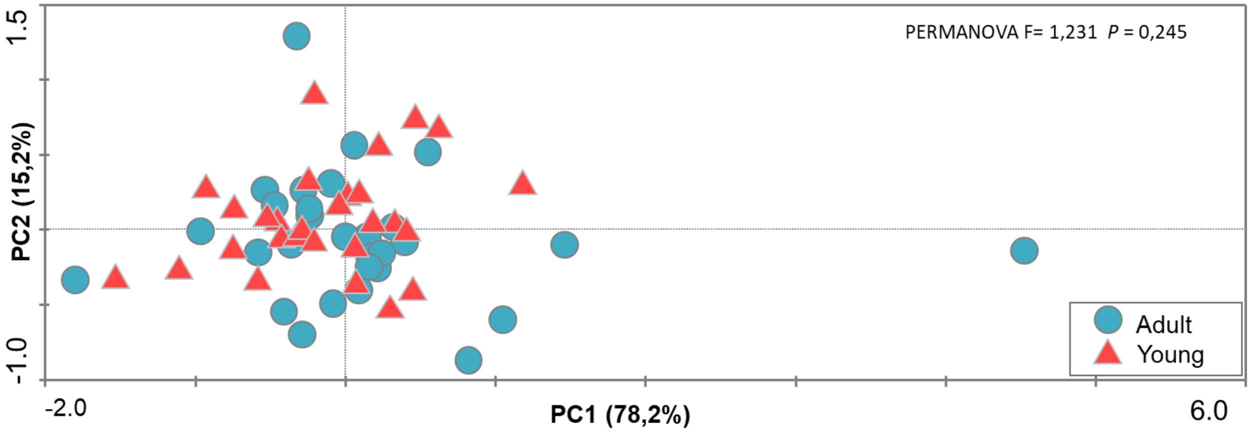

Supplementary, Figure S1. Principal Component Analysis of fecal microbial communities at genus level, in Adult and Young beef cattle from Semi-Intensive and Traditional systems.

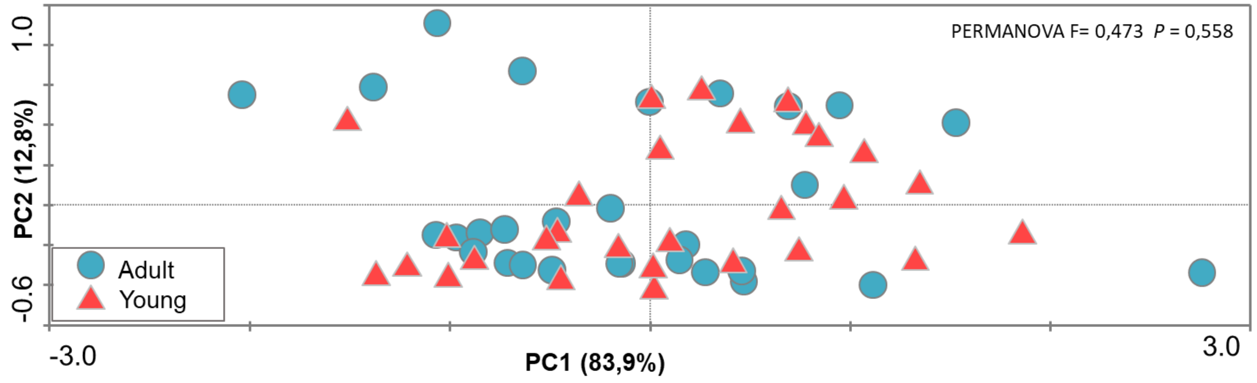

Supplementary, Figure S2. Principal Component Analysis of fecal microbial communities at function profile (level 3), in Adult and Young beef cattle from Semi-Intensive and Traditional systems.

Supplementary, Table S1. Feed and nutrient composition from diets (g/Kg of DM) of Semi-intensive (I) and Traditional (T) beef production system farms.

Farm	System	Forage					
		DM	OM	CP	NDF	ADF	MM
**1**	I	502.1	934.6	80.6	748.0	456.5	65.4
**2**	I	693.5	948.7	43.9	839.5	545.9	51.3
**3**	T	484.3	928.0	34.0	792.1	541.5	72.0
**4**	T	495.1	901.1	59.8	697.5	391.4	98.9
**5**	T	496.0	906.9	55.0	718.2	419.4	93.1
**6**	T	603.4	939.0	44.3	788.4	480.4	61.0
**Supplementary Feed**							
**1**	I	418.4	948.2	134.4	486.8	288.8	51.8
**2**	I	380.5	896.7	73.9	540.9	322.1	103.3
**5**	T	770.4	951.8	160.9	169.6	101.7	48.2

Forage (F): Farm 1: *Cynodon dactylon* cv Jiggs, *Brachiaria brizantha* cv Piatã; Farm 2: *Brachiaria brizantha cv*. Marandu; Farm 3: *Brachiaria brizantha* cv. Marandu; Farm 4: *Brachiaria brizantha* cv. Marandu; Farm5: *Brachiaria decumbens* + *Brachiaria brizantha* cv. Marandu; Farm 6: *Brachiaria brizantha* cv. Marandu. Supplementary Foods (SF): Farm 1: Maize silage + commercial feed mixture; Farm 2 (Silage + commercial feed mixture); Farm 5: Mixture (maize, soybean meal, mineral salt, and urea). Dry matter (DM), mineral matter (MM), organic matter (OM), crude protein (CP), neutral detergent fiber (NDF), acid detergent fiber (ADF)

Supplementary, Table S2. Proportion of sequences (%) affiliated to REFSEQ Database, level Family in the fecal microbiota of I (Semi-intensive) and T (Traditional) beef production systems. Bold *P*-values indicate significant diferences based on two-side Whelch's t-test corrected by benjamini-Hochberg DFR.

	**I**	**T**	***P*-values**
Desulfovibrionaceae	0.282	0.541	5.62E-18
Natranaerobiaceae	0.074	0.100	7.50E-18
unclassified (derived from Gloeobacterales)	0.021	0.035	1.79E-16
Prochlorococcaceae	0.039	0.059	2.10E-16
Herpetosiphonaceae	0.023	0.031	2.48E-16
Clostridiales Family XVII. Incertae Sedis	0.038	0.057	2.64E-16
Thermoanaerobacteraceae	0.973	1.362	3.27E-16
Nocardiaceae	0.039	0.056	3.30E-16
unclassified (derived from Chroococcales)	0.259	0.364	6.04E-16
Halanaerobiaceae	0.142	0.194	1.11E-15
Kineosporiaceae	0.012	0.018	1.18E-15
Phyllobacteriaceae	0.054	0.084	1.21E-15
Rhodospirillaceae	0.080	0.163	1.22E-15
Thermoanaerobacterales Family III. Incertae Sedis	0.790	1.138	1.24E-15
Clostridiales Family XVIII. Incertae Sedis	0.124	0.176	1.27E-15
Dictyoglomaceae	0.062	0.086	1.36E-15
Oscillochloridaceae	0.005	0.008	1.39E-15
Nostocaceae	0.076	0.104	1.68E-15
Sphaerobacteraceae	0.027	0.038	1.73E-15
unclassified (derived from Alphaproteobacteria)	0.010	0.023	1.79E-15
Desulfomicrobiaceae	0.030	0.045	1.85E-15
Beijerinckiaceae	0.011	0.017	1.89E-15
Alicyclobacillaceae	0.103	0.141	1.90E-15
Rubrobacteraceae	0.027	0.041	2.09E-15
Rhodobacteraceae	0.165	0.252	2.39E-15
Desulfohalobiaceae	0.024	0.036	2.57E-15
Halobacteroidaceae	0.048	0.063	3.17E-15
Ktedonobacteraceae	0.015	0.021	3.23E-15
Sphingomonadaceae	0.056	0.086	3.26E-15
Thermotogaceae	0.287	0.368	3.33E-15
Nocardiopsaceae	0.021	0.029	3.84E-15
Bradyrhizobiaceae	0.107	0.153	4.63E-15
Thermomicrobiaceae	0.015	0.021	5.56E-15
Micrococcaceae	0.058	0.079	5.85E-15
Erythrobacteraceae	0.017	0.027	5.98E-15
Frankiaceae	0.034	0.049	7.89E-15

Tsukamurellaceae	0.004	0.006	7.94E-15
Nitrospiraceae	0.031	0.045	8.93E-15
Chloroflexaceae	0.120	0.166	1.10E-14
Brucellaceae	0.027	0.042	1.39E-14
Hyphomonadaceae	0.035	0.057	2.15E-14
Synergistaceae	0.232	0.303	2.39E-14
Pseudonocardiaceae	0.036	0.050	2.41E-14
Xanthobacteraceae	0.026	0.040	2.41E-14
unclassified (derived from Oscillatoriales)	0.049	0.067	2.42E-14
Nocardioidaceae	0.027	0.038	2.80E-14
Thermaceae	0.065	0.091	2.82E-14
Dermacoccaceae	0.006	0.009	3.09E-14
Caulobacteraceae	0.053	0.083	4.12E-14
unclassified (derived from Thermotogales)	0.011	0.015	4.16E-14
Syntrophobacteraceae	0.051	0.077	4.26E-14
Sanguibacteraceae	0.015	0.020	4.52E-14
Hyphomicrobiaceae	0.012	0.019	4.76E-14
Rhizobiaceae	0.098	0.147	5.07E-14
Trueperaceae	0.012	0.018	5.30E-14
Methylocystaceae	0.002	0.004	5.84E-14
unclassified (derived from Rickettsiales)	0.009	0.015	6.40E-14
Glycomycetaceae	0.008	0.011	7.10E-14
Aquificaceae	0.061	0.083	8.19E-14
Nakamurellaceae	0.007	0.010	1.04E-13
unclassified (derived from Cyanobacteria)	0.013	0.017	1.05E-13
Streptosporangiaceae	0.014	0.018	1.32E-13
Deinococcaceae	0.047	0.064	1.50E-13
Paenibacillaceae	0.575	0.745	1.50E-13
Geobacteraceae	0.249	0.342	1.56E-13
Pelobacteraceae	0.125	0.169	2.35E-13
Thermodesulfobiaceae	0.022	0.026	2.41E-13
Desulfarculaceae	0.015	0.023	3.34E-13
Rickettsiaceae	0.028	0.045	3.91E-13
Geodermatophilaceae	0.006	0.008	5.15E-13
Methylobacteriaceae	0.028	0.050	5.38E-13
Desulfuromonadaceae	0.031	0.039	7.28E-13
unclassified (derived from Elusimicrobia)	0.017	0.023	8.97E-13
Syntrophaceae	0.060	0.085	9.71E-13
Acidobacteriaceae	0.033	0.052	1.10E-12
Desulfobacteraceae	0.115	0.154	1.11E-12
Bartonellaceae	0.014	0.023	1.18E-12
Catenulisporaceae	0.010	0.014	1.47E-12
Actinosynnemataceae	0.009	0.011	1.57E-12

Polyangiaceae	0.018	0.025	1.79E-12
Beutenbergiaceae	0.013	0.019	1.97E-12
Parvularculaceae	0.006	0.009	1.97E-12
Syntrophomonadaceae	0.249	0.307	2.20E-12
Desulfobulbaceae	0.064	0.089	2.75E-12
Streptomycetaceae	0.099	0.128	3.67E-12
Micromonosporaceae	0.026	0.033	3.94E-12
Dermabacteraceae	0.011	0.015	4.45E-12
Conexibacteraceae	0.014	0.019	4.81E-12
unclassified (derived from Acidobacteria)	0.036	0.052	5.14E-12
Mariprofundaceae	0.006	0.009	5.44E-12
unclassified (derived from Actinomycetales)	0.003	0.004	1.31E-11
Chlamydiaceae	0.020	0.035	1.33E-11
Acidothermaceae	0.010	0.014	1.41E-11
Myxococcaceae	0.097	0.145	2.02E-11
Hydrogenothermaceae	0.047	0.062	2.43E-11
Victivallaceae	0.132	0.583	3.10E-11
Aurantimonadaceae	0.009	0.014	3.23E-11
Anaplasmataceae	0.024	0.037	3.48E-11
Deferribacteraceae	0.078	0.102	4.69E-11
Segniliparaceae	0.002	0.003	5.17E-11
Acidimicrobiaceae	0.003	0.005	6.25E-11
Thermomonosporaceae	0.009	0.011	6.90E-11
Haliangiaceae	0.018	0.024	7.02E-11
unclassified (derived from Actinobacteria (class))	0.006	0.009	7.22E-11
Solibacteraceae	0.059	0.084	1.80E-10
Lentisphaeraceae	0.021	0.047	2.09E-10
Bdellovibrionaceae	0.023	0.032	2.65E-10
Gemmatimonadaceae	0.010	0.015	3.30E-10
Intrasporangiaceae	0.009	0.012	4.16E-10
Parachlamydiaceae	0.019	0.031	5.61E-10
Jonesiaceae	0.009	0.011	7.40E-10
Leptospiraceae	0.050	0.069	9.18E-10
Clostridiales Family XI. Incertae Sedis	0.503	0.615	9.27E-10
Microbacteriaceae	0.020	0.029	9.30E-10
Chrysiogenaceae	0.016	0.021	1.04E-09
unclassified (derived from Epsilonproteobacteria)	0.040	0.051	1.04E-09
Mycobacteriaceae	0.063	0.082	1.10E-09
unclassified (derived from Deltaproteobacteria)	0.018	0.025	1.36E-09
Peptococcaceae	1.212	1.447	1.37E-09
Planctomycetaceae	0.121	0.197	1.49E-09

Peptostreptococcaceae	0.148	0.181	2.23E-09
Brevibacteriaceae	0.010	0.012	2.27E-09
Nannocystaceae	0.007	0.011	2.30E-09
Propionibacteriaceae	0.024	0.030	2.38E-09
Nautiliaceae	0.014	0.019	2.87E-09
unclassified (derived from Proteobacteria)	0.029	0.036	3.14E-09
Waddliaceae	0.009	0.013	1.29E-08
Verrucomicrobia subdivision 3	0.019	0.040	1.47E-08
Promicromonosporaceae	0.014	0.017	2.78E-08
Methylacidiphilaceae	0.013	0.027	4.68E-08
Opitutaceae	0.074	0.136	7.61E-08
unclassified (derived from Bacillales)	0.127	0.151	1.50E-07
Gordoniaceae	0.004	0.005	1.70E-07
Fusobacteriaceae	0.799	0.899	1.96E-07
unclassified (derived from Verrucomicrobiales)	0.019	0.032	2.04E-07
Puniceicoccaceae	0.024	0.049	2.11E-07
Hydrogenophilaceae	0.016	0.020	3.20E-07
Carnobacteriaceae	0.059	0.071	3.29E-07
Acholeplasmataceae	0.131	0.172	3.95E-07
Chlorobiaceae	0.287	0.357	6.29E-07
Brachyspiraceae	0.214	0.240	7.73E-07
Corynebacteriaceae	0.100	0.129	1.18E-06
Cellulomonadaceae	0.009	0.010	1.49E-06
unclassified (derived from Rhizobiales)	0.000	0.000	1.62E-06
Ruminococcaceae	7.816	8.931	2.38E-06
Cystobacteraceae	0.014	0.017	2.45E-06
unclassified (derived from Spartobacteria)	0.024	0.054	2.68E-06
Heliobacteriaceae	0.137	0.160	3.08E-06
Leuconostocaceae	0.087	0.102	5.95E-06
Lachnospiraceae	7.143	4.947	6.46E-06
Rhodothermaceae	0.083	0.102	6.88E-06
unclassified (derived from Clostridiales)	1.631	1.234	1.13E-05
Eubacteriaceae	6.536	4.769	2.40E-05
Bacillaceae	2.145	2.414	6.03E-05
Cyclobacteriaceae	0.054	0.068	0.0001
Staphylococcaceae	0.250	0.286	0.0003
Elusimicrobiaceae	0.075	0.118	0.0003
Methylococcaceae	0.033	0.040	0.0004
Aeromonadaceae	0.235	0.063	0.0006
Acetobacteraceae	0.078	0.113	0.0007
Moritellaceae	0.019	0.006	0.0007
Psychromonadaceae	0.064	0.033	0.0007
unclassified (derived from Bacteria)	0.067	0.080	0.0007

Verrucomicrobiaceae	0.297	0.652	0.0008
Listeriaceae	0.221	0.245	0.0008
Flammeovirgaceae	0.099	0.120	0.0009
Comamonadaceae	0.110	0.124	0.0010
Ferrimonadaceae	0.023	0.010	0.0011
unclassified (derived from Flavobacteriales)	0.077	0.092	0.0012
Acidithiobacillaceae	0.012	0.014	0.0013
Vibrionaceae	0.431	0.270	0.0015
Xanthomonadaceae	0.075	0.085	0.0018
Bifidobacteriaceae	0.724	0.437	0.0018
Sphingobacteriaceae	0.492	0.586	0.0019
Alcaligenaceae	0.042	0.049	0.0019
unclassified (derived from Vibrionales)	0.004	0.003	0.0025
Colwelliaceae	0.031	0.021	0.0027
Shewanellaceae	0.277	0.193	0.0031
Pseudoalteromonadaceae	0.095	0.059	0.0032
Enterobacteriaceae	0.792	0.524	0.0034
Idiomarinaceae	0.044	0.026	0.0039
Cytophagaceae	0.686	0.799	0.0040
Francisellaceae	0.053	0.057	0.0041
Acidaminococcaceae	0.425	0.351	0.0042
Rhodocyclaceae	0.073	0.082	0.0042
Pasteurellaceae	0.396	0.282	0.0050
Aerococcaceae	0.330	0.296	0.0053
Flavobacteriaceae	1.776	2.057	0.0060
Burkholderiaceae	0.188	0.209	0.0061
unclassified (derived from Flavobacteria)	0.103	0.120	0.0075
unclassified (derived from Alteromonadales)	0.024	0.017	0.0085
Entomoplasmataceae	0.015	0.017	0.0098
unclassified (derived from Sphingobacteriales)	0.192	0.219	0.0106
Nitrosomonadaceae	0.041	0.045	0.0107
unclassified (derived from Campylobacterales)	0.005	0.006	0.0115
Alteromonadaceae	0.099	0.080	0.0142
Coriobacteriaceae	1.309	1.462	0.0207
Oceanospirillaceae	0.052	0.042	0.0222
Lactobacillaceae	0.798	0.915	0.0223
Blattabacteriaceae	0.017	0.020	0.0239
Veillonellaceae	1.049	0.751	0.0273
Thiotrichaceae	0.008	0.007	0.0419

Supplementary, Table S3. Proportion of sequences (%) affiliated to REFSEQ Database, level genus in the fecal microbiota of I (Semi-intensive) and T (Traditional) beef production systems. Bold *P*-values indicate significant diferences based on two-side Whelch's t-test corrected by benjamini-Hochberg DFR.

	I	T	*P*-values
Natranaerobius	0.07425	0.10017	1.85E-17
Desulfovibrio	0.26020	0.49715	1.75E-17
Candidatus Desulforudis	0.05597	0.07888	2.62E-16
Synechococcus	0.10410	0.16047	3.82E-16
Prochlorococcus	0.03929	0.05860	3.10E-16
Gloeobacter	0.02138	0.03465	2.94E-16
Chelativorans	0.01471	0.02272	3.43E-16
Thermaerobacter	0.03849	0.05717	4.06E-16
Nocardia	0.01312	0.02167	3.79E-16
Herpetosiphon	0.02281	0.03086	3.66E-16
Maritimibacter	0.00481	0.00730	3.61E-16
Renibacterium	0.00432	0.00685	4.75E-16
Synechocystis	0.02316	0.03394	4.79E-16
Thermosynechococcus	0.02003	0.02996	4.71E-16
Ruegeria	0.02295	0.03523	4.88E-16
Methylocella	0.00605	0.00973	4.86E-16
Thermobaculum	0.02666	0.03962	6.79E-16
Actinomyces	0.03012	0.04152	8.36E-16
Bradyrhizobium	0.03342	0.04727	8.25E-16
Parvibaculum	0.01548	0.02599	9.04E-16
Caldanaerobacter	0.33780	0.49462	9.23E-16
Halothermothrix	0.09863	0.13867	1.19E-15
Ammonifex	0.03711	0.05388	1.15E-15
Anaerotruncus	0.39060	0.60045	1.17E-15
Roseobacter	0.02083	0.03148	1.17E-15
Rhizobium	0.03950	0.06093	1.15E-15
Magnetospirillum	0.03257	0.06544	1.13E-15
Oligotropha	0.00488	0.00761	1.16E-15
Kineococcus	0.01197	0.01790	1.21E-15
Symbiobacterium	0.12445	0.17625	1.56E-15
Rhodospirillum	0.03496	0.07391	1.73E-15
Dictyoglomus	0.06238	0.08608	1.68E-15
Moorella	0.12407	0.17132	1.66E-15
Oscillochloris	0.00547	0.00833	1.71E-15
Alicyclobacillus	0.06314	0.08804	1.95E-15

Caldicellulosiruptor	0.54434	0.81930	1.95E-15
unclassified (derived from Alphaproteobacteria)	0.00585	0.01375	1.92E-15
Ethanoligenens	0.68511	1.04029	2.07E-15
Sphaerobacter	0.02675	0.03816	2.07E-15
Syntrophothermus	0.04680	0.06478	2.07E-15
Desulfomicrobium	0.02997	0.04476	2.22E-15
Xanthobacter	0.01147	0.01822	2.22E-15
Carboxydothermus	0.15605	0.21135	2.28E-15
Azospirillum	0.01244	0.02342	2.36E-15
Nostoc	0.04033	0.05173	2.42E-15
Phenylobacterium	0.00655	0.01041	2.67E-15
Rubrobacter	0.02747	0.04078	2.62E-15
Thermotoga	0.11487	0.15570	3.36E-15
Thermoanaerobacter	0.31803	0.43096	3.49E-15
Thermobispora	0.00836	0.01120	3.58E-15
Sphingomonas	0.01305	0.02039	3.52E-15
Roseovarius	0.00857	0.01263	3.69E-15
Loktanella	0.00289	0.00465	4.12E-15
Ktedonobacter	0.01470	0.02124	4.27E-15
Acetohalobium	0.04777	0.06343	4.26E-15
Aminobacterium	0.02853	0.03949	5.77E-15
Cylindrospermopsis	0.00286	0.00464	5.80E-15
Thermobifida	0.01381	0.01880	7.29E-15
unclassified (derived from Alicyclobacillaceae)	0.03977	0.05336	7.33E-15
Thermomicrobium	0.01486	0.02130	7.52E-15
Erythrobacter	0.01695	0.02723	8.45E-15
Sinorhizobium	0.02948	0.04246	1.09E-14
Frankia	0.03435	0.04898	1.11E-14
Tsukamurella	0.00416	0.00586	1.13E-14
Roseomonas	0.00401	0.00866	1.15E-14
Kocuria	0.00530	0.00738	1.14E-14
Oscillatoria	0.00434	0.00671	1.26E-14
Desulfonatronospira	0.00808	0.01123	1.45E-14
Novosphingobium	0.01113	0.01751	1.53E-14
Ochrobactrum	0.01039	0.01544	1.62E-14
Pseudovibrio	0.00518	0.00874	1.61E-14
Thermodesulfovibrio	0.02120	0.02987	1.61E-14
Oceanithermus	0.00767	0.01142	1.62E-14
Rhodomicrobium	0.00563	0.00942	1.61E-14
Sphingobium	0.00730	0.01125	1.63E-14

Desulfohalobium	0.01581	0.02440	1.83E-14
Candidatus Puniceispirillum	0.00429	0.00886	1.94E-14
Aquifex	0.02790	0.04015	2.03E-14
Dethiobacter	0.04001	0.05690	2.01E-14
Arthrobacter	0.03507	0.04807	2.08E-14
Cyanobium	0.00181	0.00302	2.25E-14
Thermus	0.02883	0.04249	2.58E-14
Pelotomaculum	0.13895	0.18297	2.60E-14
Kribbella	0.01012	0.01562	2.62E-14
unclassified (derived from Ruminococcaceae)	1.05769	1.47062	2.64E-14
Raphidiopsis	0.00179	0.00327	2.74E-14
Anaerobaculum	0.01141	0.01612	3.05E-14
Hyphomonas	0.01182	0.01892	3.26E-14
Oceanicaulis	0.00589	0.01042	3.56E-14
Caulobacter	0.03012	0.04775	3.65E-14
Roseibium	0.00258	0.00491	3.72E-14
Halanaerobium	0.04316	0.05582	3.93E-14
Roseiflexus	0.07611	0.10827	3.93E-14
Dinoroseobacter	0.00660	0.00959	4.06E-14
Anabaena	0.02758	0.03851	4.09E-14
Nitrobacter	0.01621	0.02505	4.22E-14
Saccharomonospora	0.00553	0.00758	4.81E-14
unclassified (derived from Thermotogales)	0.01064	0.01522	5.11E-14
Syntrophobacter	0.05146	0.07727	5.40E-14
Labrenzia	0.00714	0.01242	5.55E-14
Sanguibacter	0.01487	0.02030	5.73E-14
Candidatus Pelagibacter	0.00713	0.01219	5.96E-14
Oceanicola	0.00731	0.01226	6.71E-14
Truepera	0.01225	0.01846	6.90E-14
Paracoccus	0.01156	0.01608	7.15E-14
Methylosinus	0.00232	0.00427	7.59E-14
Aminomonas	0.00704	0.01049	7.84E-14
Rickettsia	0.02331	0.03958	7.97E-14
Sagittula	0.00214	0.00343	8.12E-14
Brucella	0.01680	0.02634	8.38E-14
Stackebrandtia	0.00759	0.01085	9.14E-14
Chloroflexus	0.04418	0.05743	9.56E-14
Peptoniphilus	0.11991	0.16494	9.93E-14
Kytococcus	0.00450	0.00613	1.08E-13
Zymomonas	0.01106	0.01773	1.08E-13

Azorhizobium	0.00899	0.01343	1.26E-13
Acaryochloris	0.01262	0.01667	1.33E-13
Nakamurella	0.00727	0.00956	1.32E-13
Arthrospira	0.00777	0.01159	1.49E-13
Thermosediminibacter	0.10234	0.13564	1.56E-13
Streptosporangium	0.01405	0.01772	1.66E-13
Cyanothece	0.07969	0.10099	1.80E-13
Paenibacillus	0.49790	0.65092	1.88E-13
Deinococcus	0.04663	0.06431	1.88E-13
Nocardiopsis	0.00713	0.00998	1.87E-13
Sphingopyxis	0.01100	0.01612	1.94E-13
Fervidobacterium	0.04674	0.05888	1.93E-13
Geobacter	0.24881	0.34211	1.95E-13
Maricaulis	0.01138	0.01813	1.96E-13
Epulopiscium	0.05924	0.08140	2.22E-13
Mesorhizobium	0.02162	0.03081	2.20E-13
Meiothermus	0.02814	0.03678	2.36E-13
Afipia	0.00146	0.00278	2.48E-13
Salinispora	0.01496	0.02030	2.68E-13
Pelobacter	0.12538	0.16941	2.83E-13
Coprothermobacter	0.02154	0.02596	2.93E-13
Hirschia	0.00609	0.00987	2.99E-13
Microcoleus	0.00789	0.01121	3.02E-13
Citromicrobium	0.00200	0.00306	3.00E-13
unclassified (derived from Clostridiales Family XI. Incertae Sedis)	0.03680	0.05050	3.19E-13
Rhodopseudomonas	0.05064	0.07029	3.22E-13
unclassified (derived from Chroococcales)	0.00228	0.00427	3.30E-13
Ahrensia	0.00237	0.00439	3.33E-13
Rhodobacter	0.02753	0.04147	3.34E-13
Amycolatopsis	0.00761	0.00994	3.83E-13
Desulfarculus	0.01541	0.02326	3.82E-13
Desulfatibacillum	0.04277	0.06027	3.94E-13
Acidobacterium	0.02235	0.03433	4.04E-13
Thermanaerovibrio	0.03519	0.04565	4.56E-13
Ehrlichia	0.00686	0.01102	4.81E-13
Geodermatophilus	0.00590	0.00825	5.87E-13
Methylobacterium	0.02801	0.04979	6.18E-13
Acidiphilium	0.01295	0.02169	6.88E-13
Ilyobacter	0.10770	0.13781	7.05E-13
Hoeflea	0.00256	0.00427	7.18E-13

Desulfuromonas	0.03053	0.03946	8.27E-13
Pyramidobacter	0.09903	0.12871	1.01E-12
unclassified (derived from Elusimicrobia)	0.01712	0.02350	1.02E-12
Hyphomicrobium	0.00589	0.00948	1.06E-12
Beijerinckia	0.00495	0.00735	1.09E-12
Syntrophus	0.05988	0.08474	1.10E-12
Neorickettsia	0.00258	0.00481	1.14E-12
Saccharopolyspora	0.01496	0.02132	1.28E-12
Bartonella	0.01374	0.02332	1.36E-12
Rhodococcus	0.02603	0.03389	1.43E-12
Thermocrinis	0.00642	0.00922	1.54E-12
Trichodesmium	0.02267	0.02890	1.57E-12
Nitrospira	0.00995	0.01455	1.62E-12
Catenulispora	0.01020	0.01389	1.67E-12
Actinosynnema	0.00866	0.01125	1.80E-12
Sorangium	0.01787	0.02470	2.06E-12
Thermoanaerobacterium	0.14369	0.18310	2.16E-12
Lyngbya	0.00644	0.00887	2.18E-12
Beutenbergia	0.01261	0.01880	2.26E-12
Parvularcula	0.00608	0.00919	2.28E-12
Sulfitobacter	0.00422	0.00647	2.35E-12
Desulfotalea	0.04810	0.06698	2.65E-12
Persephonella	0.01429	0.01886	2.64E-12
Brevundimonas	0.00731	0.01195	2.93E-12
Nodularia	0.00390	0.00580	3.32E-12
Candidatus Cloacamonas	0.01169	0.01921	3.57E-12
unclassified (derived from Rickettsiales)	0.00158	0.00312	3.96E-12
Thermosipho	0.06236	0.07844	4.05E-12
Streptomyces	0.09921	0.12775	4.17E-12
Jannaschia	0.00735	0.01126	4.32E-12
Phaeobacter	0.00152	0.00213	4.69E-12
Lawsonia	0.02166	0.04381	4.70E-12
Brachybacterium	0.01052	0.01455	5.07E-12
Chlamydophila	0.01276	0.02127	5.24E-12
Conexibacter	0.01417	0.01928	5.49E-12
Kosmotoga	0.02398	0.03072	5.54E-12
Candidatus Koribacter	0.03640	0.05197	5.86E-12
Mariprofundus	0.00646	0.00909	6.24E-12
Desulfococcus	0.04143	0.05476	7.73E-12
Citreicella	0.00187	0.00291	8.70E-12
Thermincola	0.10387	0.13536	1.03E-11

Micrococcus	0.00464	0.00608	1.20E-11
Desulfurivibrio	0.01591	0.02243	1.38E-11
Deferribacter	0.02578	0.03474	1.40E-11
Myxococcus	0.03293	0.04770	1.39E-11
unclassified (derived from Rhodobacteraceae)	0.00450	0.00684	1.39E-11
Tropheryma	0.00299	0.00427	1.45E-11
Acidothermus	0.01045	0.01418	1.59E-11
Asticcacaulis	0.00942	0.01323	1.74E-11
Terriglobus	0.01108	0.01729	2.03E-11
Dermacoccus	0.00162	0.00237	2.67E-11
Alkaliphilus	0.70181	0.87411	2.81E-11
Hydrogenivirga	0.00769	0.01072	2.84E-11
Anaeromyxobacter	0.06452	0.09753	2.96E-11
Oceanibulbus	0.00114	0.00190	3.43E-11
Victivallis	0.13180	0.58324	3.47E-11
Dethiosulfovibrio	0.03631	0.04567	3.76E-11
Segniliparus	0.00228	0.00305	5.98E-11
Nocardioides	0.01533	0.02045	6.94E-11
Acidimicrobium	0.00325	0.00510	7.22E-11
Calditerrivibrio	0.01891	0.02687	7.34E-11
Thermomonospora	0.00930	0.01141	7.98E-11
Haliangium	0.01779	0.02445	8.17E-11
unclassified (derived from Actinobacteria (class))	0.00587	0.00885	8.44E-11
Chlamydia	0.00754	0.01372	9.71E-11
Desulfobacterium	0.03067	0.03870	1.22E-10
Fulvimarina	0.00250	0.00440	1.32E-10
Brevibacillus	0.07680	0.09424	1.54E-10
Aurantimonas	0.00616	0.00965	1.65E-10
Nautilia	0.00992	0.01320	1.78E-10
Janibacter	0.00511	0.00685	1.84E-10
Candidatus Solibacter	0.05856	0.08415	2.05E-10
Sulfurihydrogenibium	0.03272	0.04267	2.11E-10
Thalassobium	0.00153	0.00273	2.36E-10
Lentisphaera	0.02130	0.04729	2.37E-10
Granulibacter	0.01186	0.01962	2.76E-10
Bdellovibrio	0.02340	0.03210	3.01E-10
Pelagibaca	0.00203	0.00303	3.21E-10
Planctomyces	0.03920	0.06693	3.26E-10
Anaerofustis	0.11970	0.14884	3.33E-10
Gemmatimonas	0.01039	0.01514	3.72E-10

Candidatus Protochlamydia	0.01389	0.02419	4.40E-10
Nitratiruptor	0.01807	0.02467	5.68E-10
Blastopirellula	0.02103	0.03455	7.92E-10
Jonesia	0.00921	0.01113	8.42E-10
Peptostreptococcus	0.10233	0.12548	1.01E-09
Leptospira	0.04969	0.06871	1.05E-09
Desulfurispirillum	0.01577	0.02102	1.21E-09
Clavibacter	0.01237	0.01776	1.28E-09
Mycobacterium	0.06286	0.08209	1.30E-09
unclassified (derived from Deltaproteobacteria)	0.01759	0.02456	1.61E-09
Leifsonia	0.00770	0.01141	1.78E-09
Anaplasma	0.00297	0.00496	2.22E-09
Desulfotomaculum	0.34051	0.40501	2.61E-09
Brevibacterium	0.01029	0.01201	2.72E-09
Plesiocystis	0.00750	0.01071	2.77E-09
Propionibacterium	0.02449	0.03042	2.88E-09
Sebaldella	0.11258	0.13193	3.20E-09
Pirellula	0.01364	0.02263	3.93E-09
Magnetococcus	0.02853	0.03642	3.92E-09
Chlorobaculum	0.04938	0.06658	3.94E-09
Hydrogenobaculum	0.01142	0.01392	4.88E-09
Denitrovibrio	0.03291	0.04064	5.44E-09
Starkeya	0.00548	0.00848	6.66E-09
Gemmata	0.00768	0.01182	9.25E-09
Rothia	0.00863	0.01079	9.32E-09
unclassified (derived from Opitutaceae)	0.02342	0.04921	1.27E-08
Waddlia	0.00912	0.01343	1.54E-08
unclassified (derived from Verrucomicrobia subdivision 3)	0.01945	0.03963	1.76E-08
Intrasporangium	0.00376	0.00517	1.80E-08
Wolbachia	0.01112	0.01625	1.95E-08
Hydrogenobacter	0.00713	0.00897	2.11E-08
Ketogulonicigenium	0.00438	0.00569	2.96E-08
Streptobacillus	0.03824	0.04470	3.16E-08
Rhodopirellula	0.03939	0.06102	3.19E-08
Anaerococcus	0.20490	0.24430	3.23E-08
Xylanimonas	0.01436	0.01676	3.25E-08
Parachlamydia	0.00474	0.00703	3.89E-08
Sulfurovum	0.02167	0.02682	3.92E-08

Verminephrobacter	0.01153	0.01356	4.69E-08
Catonella	0.01653	0.02060	4.70E-08
Methylacidiphilum	0.01282	0.02671	5.40E-08
Acholeplasma	0.10380	0.13895	7.04E-08
Gemella	0.02170	0.02752	7.52E-08
Agrobacterium	0.02697	0.04030	1.05E-07
Oceanobacillus	0.09747	0.11640	1.29E-07
Jonquetella	0.01429	0.01714	1.96E-07
Gordonia	0.00431	0.00538	1.97E-07
unclassified (derived from Verrucomicrobiales)	0.01853	0.03212	2.39E-07
Coraliomargarita	0.02352	0.04936	2.48E-07
Opitutus	0.05073	0.08642	3.67E-07
Thiobacillus	0.01557	0.01991	3.76E-07
Exiguobacterium	0.10567	0.12386	4.24E-07
Carnobacterium	0.02015	0.02421	4.37E-07
Syntrophomonas	0.16259	0.18490	4.78E-07
Filifactor	0.04592	0.05545	4.78E-07
Granulicatella	0.03928	0.04686	5.24E-07
Acetivibrio	0.19869	0.24161	5.76E-07
Brachyspira	0.21447	0.24011	9.13E-07
Candidatus Liberibacter	0.00232	0.00326	1.12E-06
Subdoligranulum	0.50496	0.60139	1.20E-06
Halorhodospira	0.01271	0.01634	1.19E-06
Corynebacterium	0.10025	0.12938	1.39E-06
Cellulomonas	0.00852	0.01037	1.75E-06
Candidatus Hodgkinia	0.00003	0.00008	1.91E-06
Coprococcus	0.73706	0.48659	2.11E-06
Gardnerella	0.05354	0.03900	2.18E-06
Blautia	0.96979	0.66239	2.44E-06
Stigmatella	0.01396	0.01679	2.90E-06
Pelodictyon	0.05271	0.06325	2.92E-06
Chthoniobacter	0.02399	0.05430	3.17E-06
Methylococcus	0.02610	0.03326	3.49E-06
Heliobacterium	0.13737	0.16010	3.65E-06
Chloroherpeton	0.03759	0.04746	3.72E-06
Rhodothermus	0.04836	0.06031	3.95E-06
Chlorobium	0.13373	0.16353	4.03E-06
Geobacillus	0.34736	0.39898	4.07E-06
Alicycliphilus	0.00335	0.00418	4.66E-06
Weissella	0.00716	0.00867	5.00E-06
Caminibacter	0.00457	0.00614	5.18E-06

Oenococcus	0.02585	0.03030	5.40E-06
Collinsella	0.13331	0.16166	6.71E-06
Roseburia	2.01643	1.12872	9.74E-06
Verrucomicrobium	0.02055	0.04571	9.85E-06
Micromonospora	0.01093	0.01264	1.07E-05
Fusobacterium	0.41161	0.45179	1.22E-05
Prosthecochloris	0.01337	0.01651	1.23E-05
Leuconostoc	0.05404	0.06308	1.30E-05
Dorea	0.68560	0.47325	1.33E-05
Microcystis	0.01641	0.01920	1.99E-05
Eubacterium	6.35144	4.54976	2.00E-05
Anaerostipes	0.25587	0.17267	1.99E-05
Microscilla	0.05581	0.07218	2.14E-05
Polaromonas	0.02273	0.02626	2.30E-05
Salinibacter	0.03453	0.04215	2.93E-05
Petrotoga	0.03888	0.04383	3.14E-05
Orientia	0.00421	0.00553	3.21E-05
Butyrivibrio	1.84147	1.36717	4.07E-05
Desulfitobacterium	0.57285	0.64505	4.32E-05
Sulfurimonas	0.02297	0.02791	4.61E-05
unclassified (derived from Lachnospiraceae)	0.87233	0.65014	5.22E-05
Faecalibacterium	0.88169	1.01921	5.92E-05
unclassified (derived from Clostridiales)	0.60228	0.49026	7.80E-05
Bacillus	1.54476	1.72973	0.0001
Wolinella	0.01774	0.02211	0.0001
Algoriphagus	0.05396	0.06768	0.0002
Xanthomonas	0.04369	0.05109	0.0002
Staphylococcus	0.21688	0.24934	0.0002
Gluconobacter	0.01612	0.02280	0.0003
Sulfuricurvum	0.00693	0.00867	0.0003
Elusimicrobium	0.07507	0.11840	0.0003
Mucilaginibacter	0.05146	0.06412	0.0003
Polynucleobacter	0.01237	0.01487	0.0004
Herminiimonas	0.01317	0.01542	0.0004
Candidatus Sulcia	0.00697	0.00835	0.0004
Tolumonas	0.09058	0.01962	0.0005
Candidatus Amoebophilus	0.03700	0.04548	0.0005
Methylibium	0.00962	0.01137	0.0007
Aeromonas	0.14490	0.04332	0.0007
Moritella	0.01862	0.00579	0.0008

Candidatus Phytoplasma	0.02729	0.03350	0.0008
Psychromonas	0.06363	0.03277	0.0008
Ralstonia	0.02607	0.03026	0.0008
Dechloromonas	0.02462	0.02839	0.0009
Listeria	0.22143	0.24514	0.0009
unclassified (derived from Vibrionaceae)	0.01378	0.00482	0.0010
Mannheimia	0.01994	0.01356	0.0010
Marivirga	0.09894	0.12048	0.0010
unclassified (derived from Alteromonadales)	0.01111	0.00412	0.0010
Akkermansia	0.27689	0.60659	0.0011
Finegoldia	0.14136	0.15561	0.0012
Candidatus Hamiltonella	0.00549	0.00299	0.0012
Ferrimonas	0.02278	0.01021	0.0012
Cytophaga	0.19816	0.23926	0.0013
Sphingobacterium	0.09103	0.10996	0.0013
Aggregatibacter	0.03005	0.01685	0.0013
Glaciecola	0.00008	0.00004	0.0013
Candidatus Regiella	0.00331	0.00116	0.0014
Acidithiobacillus	0.01196	0.01420	0.0015
Macrococcus	0.03326	0.03711	0.0015
unclassified (derived from Flavobacteriales)	0.06972	0.08374	0.0015
Vibrio	0.25125	0.15682	0.0016
Bordetella	0.03166	0.03702	0.0016
Nitrosospira	0.01198	0.01383	0.0016
Dickeya	0.03011	0.01901	0.0016
Aliivibrio	0.05893	0.03748	0.0018
Sulfurospirillum	0.01345	0.01781	0.0018
Chromobacterium	0.02360	0.02711	0.0018
Pediococcus	0.04578	0.05080	0.0018
Thauera	0.01038	0.01189	0.0020
Photobacterium	0.10672	0.07043	0.0021
Alteromonas	0.02815	0.01662	0.0021
Kordia	0.03571	0.04273	0.0022
Abiotrophia	0.29916	0.26262	0.0022
Aromatoleum	0.02048	0.02308	0.0023
Gramella	0.18230	0.21594	0.0023
Actinobacillus	0.11638	0.08202	0.0025
Xenorhabdus	0.01610	0.00928	0.0025
Edwardsiella	0.02570	0.01517	0.0026
Bifidobacterium	0.64184	0.37503	0.0027

Turicibacter	0.14148	0.11130	0.0027
unclassified (derived from Vibrionales)	0.00426	0.00257	0.0028
Providencia	0.02845	0.01743	0.0029
Colwellia	0.03099	0.02109	0.0030
Photorhabdus	0.03992	0.02592	0.0031
Shewanella	0.27671	0.19305	0.0033
Candidatus Zinderia	0.00007	0.00010	0.0034
Pseudoalteromonas	0.09470	0.05913	0.0035
Pedobacter	0.34911	0.41231	0.0035
Croceibacter	0.08443	0.10047	0.0035
Pasteurella	0.03888	0.02341	0.0036
Proteus	0.03567	0.02150	0.0040
Aerococcus	0.01816	0.02021	0.0040
Flavobacterium	0.39774	0.46872	0.0040
Psychroflexus	0.02815	0.03327	0.0040
Sodalis	0.01067	0.00634	0.0041
Idiomarina	0.04440	0.02570	0.0042
Scardovia	0.01381	0.01120	0.0044
Francisella	0.05294	0.05749	0.0044
Acidaminococcus	0.42537	0.35099	0.0046
Enterobacter	0.03831	0.02702	0.0047
Comamonas	0.00722	0.00828	0.0056
Pantoea	0.02807	0.01862	0.0057
Shuttleworthia	0.14254	0.12645	0.0057
Salmonella	0.04827	0.03564	0.0058
Erwinia	0.01999	0.01296	0.0059
Cupriavidus	0.03779	0.04231	0.0059
Maribacter	0.11165	0.12910	0.0062
Bermanella	0.00748	0.00477	0.0063
Dokdonia	0.06653	0.07730	0.0064
Arcobacter	0.03337	0.03745	0.0067
Achromobacter	0.01074	0.01207	0.0068
Methylobacillus	0.01516	0.01714	0.0071
Neptuniibacter	0.00740	0.00513	0.0074
Pseudoramibacter	0.06508	0.07083	0.0074
Catenibacterium	0.18780	0.14652	0.0075
Chryseobacterium	0.04512	0.05203	0.0076
Cronobacter	0.02639	0.01850	0.0076
Haemophilus	0.11102	0.07928	0.0079
Pectobacterium	0.04073	0.03018	0.0080
unclassified (derived from Flavobacteria)	0.10313	0.11951	0.0080

Laribacter	0.01011	0.01173	0.0085
Janthinobacterium	0.01284	0.01444	0.0086
Polaribacter	0.14002	0.16147	0.0087
Octadecabacter	0.00369	0.00432	0.0091
Acidovorax	0.03205	0.03530	0.0095
Spirosoma	0.16825	0.19195	0.0098
Mitsuokella	0.19513	0.07625	0.0104
Mesoplasma	0.01516	0.01745	0.0104
Chitinophaga	0.19201	0.21935	0.0113
unclassified (derived from Campylobacterales)	0.00475	0.00556	0.0123
Serratia	0.03614	0.02807	0.0127
Yersinia	0.07386	0.05550	0.0132
Burkholderia	0.10674	0.11659	0.0134
Selenomonas	0.37343	0.19702	0.0135
Leeuwenhoekiella	0.09850	0.11222	0.0135
Zunongwangia	0.10616	0.11934	0.0143
Capnocytophaga	0.24560	0.27978	0.0145
Enhydrobacter	0.00523	0.00427	0.0145
Leptospirillum	0.00013	0.00008	0.0148
Coprobacillus	0.12498	0.09860	0.0153
Listonella	0.00004	0.00001	0.0157
Robiginitalea	0.09216	0.10488	0.0166
unclassified (derived from Flavobacteriaceae)	0.06891	0.07771	0.0168
Stenotrophomonas	0.01512	0.01699	0.0170
Buchnera	0.01329	0.00919	0.0172
Dyadobacter	0.15370	0.17434	0.0180
Parascardovia	0.01443	0.01197	0.0189
Variovorax	0.00621	0.00697	0.0199
Tetragenococcus	0.00018	0.00013	0.0201
Erysipelothrix	0.03454	0.03854	0.0204
unclassified (derived from Proteobacteria)	0.00006	0.00004	0.0209
Oribacterium	0.29381	0.26932	0.0210
Cellvibrio	0.02459	0.02167	0.0229
Lysinibacillus	0.08079	0.08908	0.0237
Slackia	0.45246	0.50078	0.0241
Blattabacterium	0.01740	0.01950	0.0249
Riemerella	0.07254	0.08155	0.0256
Wigglesworthia	0.00231	0.00160	0.0258
Dehalogenimonas	0.01577	0.01708	0.0274
Bulleidia	0.04888	0.05339	0.0281

Atopobium	0.21438	0.24269	0.0283
Lactobacillus	0.75205	0.86449	0.0285
Cellulosilyticum	0.28133	0.25199	0.0291
Aeromicrobium	0.00188	0.00209	0.0292
Nitrosomonas	0.02857	0.03088	0.0329
Anoxybacillus	0.07477	0.07937	0.0353
Crocosphaera	0.01149	0.01226	0.0361
Delftia	0.00975	0.01074	0.0405
Beggiatoa	0.00755	0.00675	0.0429
Leadbetterella	0.10961	0.12137	0.0431
Collimonas	0.00006	0.00000	0.0449
unclassified (derived from Erysipelotrichaceae)	0.73384	0.65857	0.0470
Klebsiella	0.03302	0.02689	0.0472
Kangiella	0.01705	0.01421	0.0484
Olsenella	0.12915	0.14541	0.0486

Supplementary, Table S4. Proportion of sequences (%) affiliated to KEGG Database, level 3 in the fecal microbiota of I (Semi-intensive) and T (Traditional) beef production systems. Bold *P*-values indicate significant diferences based on two-side Whelch's t-test corrected by benjamini-Hochberg DFR.

	I	T	*P*-values
00660 C5-Branched dibasic acid metabolism [PATH:ko00660]	0.0485	0.0870	4.99E-20
03010 Ribosome [PATH:ko03010]	4.4783	5.0311	1.56E-14
03020 RNA polymerase [PATH:ko03020]	2.6124	2.9852	1.70E-14
03440 Homologous recombination [PATH:ko03440]	1.6456	1.8317	3.09E-13
00030 Pentose phosphate pathway [PATH:ko00030]	1.3756	1.5230	5.27E-13
00970 Aminoacyl-tRNA biosynthesis [PATH:ko00970]	7.9864	8.8438	1.17E-12
00010 Glycolysis / Gluconeogenesis [PATH:ko00010]	2.2805	2.3901	1.63E-12
00360 Phenylalanine metabolism [PATH:ko00360]	0.3807	0.4461	1.15E-11
00620 Pyruvate metabolism [PATH:ko00620]	1.7940	1.9728	1.82E-11
00400 Phenylalanine, tyrosine and tryptophan biosynthesis [PATH:ko00400]	1.0893	0.8952	3.52E-11
03030 DNA replication [PATH:ko03030]	2.6212	2.9692	3.52E-11
00311 Penicillin and cephalosporin biosynthesis [PATH:ko00311]	0.0075	0.0123	4.41E-11
00720 Carbon fixation pathways in prokaryotes [PATH:ko00720]	0.4381	0.5182	4.69E-11
00785 Lipoic acid metabolism [PATH:ko00785]	0.0330	0.0502	4.78E-11
00250 Alanine, aspartate and glutamate metabolism [PATH:ko00250]	5.1536	4.0797	4.86E-11
03420 Nucleotide excision repair [PATH:ko03420]	2.0301	2.2142	5.07E-11
04112 Cell cycle - Caulobacter [PATH:ko04112]	1.9213	2.0672	6.11E-11
00430 Taurine and hypotaurine metabolism [PATH:ko00430]	0.0006	0.0018	8.68E-11
00760 Nicotinate and nicotinamide metabolism [PATH:ko00760]	1.0954	1.0136	1.36E-10
03430 Mismatch repair [PATH:ko03430]	0.6754	0.7529	1.39E-10
00061 Fatty acid biosynthesis [PATH:ko00061]	0.7606	0.6224	4.33E-10
00562 Inositol phosphate metabolism [PATH:ko00562]	0.0301	0.0467	4.37E-10
00310 Lysine degradation [PATH:ko00310]	0.0575	0.0809	4.76E-10
03060 Protein export [PATH:ko03060]	0.1625	0.1425	7.41E-10
00520 Amino sugar and nucleotide sugar metabolism [PATH:ko00520]	1.7448	1.8732	8.72E-10
00511 Other glycan degradation [PATH:ko00511]	0.2415	0.3584	8.75E-10
02020 Two-component system [PATH:ko02020]	1.6831	1.3651	1.47E-09
00290 Valine, leucine and isoleucine biosynthesis [PATH:ko00290]	1.3087	1.0246	1.52E-09
00627 Aminobenzoate degradation [PATH:ko00627]	0.0077	0.0110	1.52E-09
00740 Riboflavin metabolism [PATH:ko00740]	0.2923	0.2309	3.18E-09

00960 Tropane, piperidine and pyridine alkaloid biosynthesis [PATH:ko00960]	0.0075	0.0100	7.00E-09
00770 Pantothenate and CoA biosynthesis [PATH:ko00770]	0.4978	0.4342	1.26E-08
00440 Phosphonate and phosphinate metabolism [PATH:ko00440]	0.0690	0.0420	1.41E-08
00240 Pyrimidine metabolism [PATH:ko00240]	2.0752	1.9537	1.84E-08
00500 Starch and sucrose metabolism [PATH:ko00500]	2.0293	1.8493	2.32E-08
03070 Bacterial secretion system [PATH:ko03070]	1.8993	2.0592	3.64E-08
00040 Pentose and glucuronate interconversions [PATH:ko00040]	1.0910	1.3043	8.23E-08
03013 RNA transport [PATH:ko03013]	0.2029	0.1556	1.09E-07
00920 Sulfur metabolism [PATH:ko00920]	0.0713	0.0296	1.51E-07
00900 Terpenoid backbone biosynthesis [PATH:ko00900]	1.1685	1.2455	1.66E-07
00730 Thiamine metabolism [PATH:ko00730]	0.6188	0.5572	1.69E-07
00600 SphingoLipid Metabolism [PATH:ko00600]	0.0006	0.0015	1.96E-07
02060 Phosphotransferase system (PTS) [PATH:ko02060]	0.3267	0.1078	2.11E-07
03018 RNA degradation [PATH:ko03018]	2.4643	2.5940	2.55E-07
00312 beta-Lactam resistance [PATH:ko00312]	0.0007	0.0001	3.03E-07
00780 Biotin metabolism [PATH:ko00780]	0.1908	0.1442	3.22E-07
02030 Bacterial chemotaxis [PATH:ko02030]	0.6917	0.2652	5.02E-07
00908 Zeatin biosynthesis [PATH:ko00908]	0.1582	0.1742	5.33E-07
00362 Benzoate degradation [PATH:ko00362]	0.0101	0.0054	7.82E-07
00270 Cysteine and methionine metabolism [PATH:ko00270]	2.2992	2.1787	1.34E-06
00513 Various types of N-glycan biosynthesis [PATH:ko00513]	0.0002	0.0005	1.43E-06
00750 Vitamin B6 metabolism [PATH:ko00750]	0.2635	0.2254	1.66E-06
02040 Flagellar assembly [PATH:ko02040]	0.4960	0.2224	1.98E-06
04151 PI3K-Akt signaling pathway [PATH:ko04151]	0.3899	0.4670	2.26E-06
00521 Streptomycin biosynthesis [PATH:ko00521]	0.8625	0.7992	2.52E-06
00361 Chlorocyclohexane and chlorobenzene degradation [PATH:ko00361]	0.0079	0.0098	2.88E-06
00910 Nitrogen metabolism [PATH:ko00910]	0.0808	0.0392	3.16E-06
00260 Glycine, serine and threonine metabolism [PATH:ko00260]	3.5176	3.7339	3.90E-06
00062 Fatty acid elongation [PATH:ko00062]	0.0011	0.0006	3.93E-06
00790 Folate biosynthesis [PATH:ko00790]	0.2397	0.2174	3.94E-06
00130 Ubiquinone and other terpenoid-quinone biosynthesis [PATH:ko00130]	0.1882	0.1420	1.49E-05
00650 Butanoate metabolism [PATH:ko00650]	0.1442	0.1226	1.65E-05
00860 Porphyrin and chlorophyll metabolism [PATH:ko00860]	0.8233	0.6561	2.41E-05
00906 Carotenoid biosynthesis [PATH:ko00906]	0.0011	0.0017	2.44E-05
04011 MAPK signaling pathway - yeast [PATH:ko04011]	0.0315	0.0254	4.22E-05
00640 Propanoate metabolism [PATH:ko00640]	0.0363	0.0438	4.46E-05
00330 Arginine and proline metabolism [PATH:ko00330]	1.8146	1.9194	5.06E-05
00625 Chloroalkane and chloroalkene degradation [PATH:ko00625]	0.0005	0.0012	5.18E-05
04141 Protein processing in endoplasmic reticulum [PATH:ko04141]	0.0661	0.0493	5.71E-05
00280 Valine, leucine and isoleucine degradation [PATH:ko00280]	0.7146	0.7986	6.19E-05

00340 Histidine metabolism [PATH:ko00340]	1.2971	1.3556	6.63E-05
03008 Ribosome biogenesis in eukaryotes [PATH:ko03008]	0.0094	0.0039	7.02E-05
00941 Flavonoid biosynthesis [PATH:ko00941]	0.0058	0.0038	7.36E-05
00100 Steroid biosynthesis [PATH:ko00100]	0.0009	0.0012	7.73E-05
00300 Lysine biosynthesis [PATH:ko00300]	1.8062	1.7597	7.92E-05
00531 Glycosaminoglycan degradation [PATH:ko00531]	0.0306	0.0409	7.93E-05
00590 Arachidonic acid metabolism [PATH:ko00590]	0.0361	0.0246	9.44E-05
02010 ABC transporters [PATH:ko02010]	5.5802	4.8620	9.88E-05
00630 Glyoxylate and dicarboxylate metabolism [PATH:ko00630]	0.3157	0.2726	0.0001
00071 Fatty acid metabolism [PATH:ko00071]	0.0151	0.0203	0.0002
00363 Bisphenol degradation [PATH:ko00363]	0.0005	0.0001	0.0003
00621 Dioxin degradation [PATH:ko00621]	0.0003	0.0001	0.0003
03022 Basal transcription factors [PATH:ko03022]	0.0166	0.0083	0.0003
04142 Lysosome [PATH:ko04142]	0.3374	0.4071	0.0003
00565 Ether Lipid Metabolism [PATH:ko00565]	0.0004	0.0008	0.0003
00051 Fructose and mannose metabolism [PATH:ko00051]	1.1631	1.2834	0.0004
01040 Biosynthesis of unsaturated fatty acids [PATH:ko01040]	0.0043	0.0014	0.0004
03050 Proteasome [PATH:ko03050]	0.0294	0.0194	0.0005
03015 mRNA surveillance pathway [PATH:ko03015]	0.0175	0.0095	0.0008
03410 Base excision repair [PATH:ko03410]	0.7674	0.7879	0.0009
04020 Calcium signaling pathway [PATH:ko04020]	0.0050	0.0064	0.0009
00190 Oxidative phosphorylation [PATH:ko00190]	2.0233	2.0888	0.0012
00591 Linoleic acid metabolism [PATH:ko00591]	0.0001	0.0004	0.0013
03450 Non-homologous end-joining [PATH:ko03450]	0.0002	0.0004	0.0017
00680 Methane metabolism [PATH:ko00680]	0.9303	0.7020	0.0018
00281 Geraniol degradation [PATH:ko00281]	0.0591	0.0695	0.0021
04630 Jak-STAT signaling pathway [PATH:ko04630]	0.0006	0.0010	0.0031
00480 Glutathione metabolism [PATH:ko00480]	0.2835	0.2638	0.0043
00230 Purine metabolism [PATH:ko00230]	4.1996	4.2812	0.0049
00140 Steroid hormone biosynthesis [PATH:ko00140]	0.0188	0.0161	0.0050
00351 1,1,1-Trichloro-2,2-bis(4-chlorophenyl)ethane (DDT) degradation [PATH:ko00351]	0.0000	0.0000	0.0051
00196 Photosynthesis - antenna proteins [PATH:ko00196]	0.0003	0.0080	0.0123
00550 Peptidoglycan biosynthesis [PATH:ko00550]	1.5102	1.4772	0.0133
00622 Xylene degradation [PATH:ko00622]	0.0001	0.0002	0.0281
00966 Glucosinolate biosynthesis [PATH:ko00966]	0.0000	0.0001	0.0319
